# Surface Patterning of Metal Zinc Electrode with an In-Region Zincophilic Interface for High-Rate and Long-Cycle-Life Zinc Metal Anode

**DOI:** 10.1007/s40820-024-01327-2

**Published:** 2024-02-09

**Authors:** Tian Wang, Qiao Xi, Kai Yao, Yuhang Liu, Hao Fu, Venkata Siva Kavarthapu, Jun Kyu Lee, Shaocong Tang, Dina Fattakhova-Rohlfing, Wei Ai, Jae Su Yu

**Affiliations:** 1 Department of Electronics and Information Convergence Engineering, Institute for Wearable Convergence Electronics, Kyung Hee University, Yongin-si, Gyeonggi-do 17104 Republic of Korea; 2 Frontiers Science Center for Flexible Electronics (FSCFE) and Shaanxi Institute of Flexible Electronics (SIFE), Northwestern Polytechnical University (NPU), 127 West Youyi Road, Xi’an, 710072 People’s Republic of China; 3 Institute of Energy and Climate Research: Materials Synthesis and Processing (IEK-1), Forschungszentrum Jülich GmbH, 52425 Jülich, Germany; 4 School of Chemical Engineering, Sungkyunkwan University, 2066 Seobu-Ro, Jangan-Gu, Suwon-si, Gyeonggi-do Republic of Korea

**Keywords:** Zn metal anode, Surface patterning, Directional Zn deposition, Aqueous Zn-I_2_ batteries

## Abstract

The undesirable dendrite growth induced by non-planar zinc (Zn) deposition and low Coulombic efficiency resulting from severe side reactions have been long-standing challenges for metallic Zn anodes and substantially impede the practical application of rechargeable aqueous Zn metal batteries (ZMBs). Herein, we present a strategy for achieving a high-rate and long-cycle-life Zn metal anode by patterning Zn foil surfaces and endowing a Zn-Indium (Zn-In) interface in the microchannels. The accumulation of electrons in the microchannel and the zincophilicity of the Zn-In interface promote preferential heteroepitaxial Zn deposition in the microchannel region and enhance the tolerance of the electrode at high current densities. Meanwhile, electron aggregation accelerates the dissolution of non-(002) plane Zn atoms on the array surface, thereby directing the subsequent homoepitaxial Zn deposition on the array surface. Consequently, the planar dendrite-free Zn deposition and long-term cycling stability are achieved (5,050 h at 10.0 mA cm^−2^ and 27,000 cycles at 20.0 mA cm^−2^). Furthermore, a Zn/I_2_ full cell assembled by pairing with such an anode can maintain good stability for 3,500 cycles at 5.0 C, demonstrating the application potential of the as-prepared ZnIn anode for high-performance aqueous ZMBs.

**Supplementary Information:**

The online version contains supplementary material available at 10.1007/s40820-024-01327-2.

## Highlights

- A stable Zn anode was obtained by patterning Zn foil surfaces and endowing a zincphilic interface in microchannels.
- The accumulation of electrons in the microchannel and the zincphilic interface promoted preferential heteroepitaxial Zn deposition in the microchannel region and subsequent homoepitaxial Zn deposition on the array surface.
- The Zn symmetrical cells could undergo repeated plating/stripping for more than 25,000 cycles at the current densities of 10 and 20 mA cm^−2^.

**Supplementary Information:**

The online version contains supplementary material available at 10.1007/s40820-024-01327-2.

## Graphical abstract

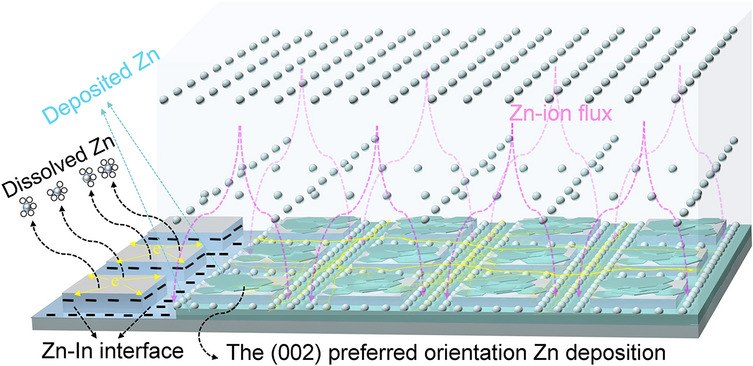

## Introduction

The ever-growing pursuit of safer and more economical energy storage systems than lithium-ion batteries (LIBs) is a critical path toward a sustainable future [1–4]. As a promising alternative beyond LIBs, aqueous batteries have been considered the next-generation large-scale energy storage devices due to their advantage in low cost and environmental friendliness [5, 6]. Among them, rechargeable aqueous zinc (Zn) metal batteries (ZMBs) have garnered considerable research interest owing to the merits of the Zn metal anode, including high theoretical capacity (820 mAh g^−1^), low redox potential (− 0.762 V vs. standard hydrogen electrode), abundance, and intrinsic safety [7, 8]. Despite its potential benefits, the implementation of metallic Zn anodes in mild electrolytes is impeded by several fundamental obstacles, such as dendrite growth induced by non-planar Zn deposition and low Coulombic efficiency (CE) due to the hydrogen evolution reaction (HER) and chemical corrosion, making the practical application of ZMBs challenging [9–12].

The stability of the electrode/electrolyte interface is considered a critical factor in determining the Zn deposition behavior and enhancing the CE of rechargeable ZMBs [13–16]. Modulating effectively and sustainably the Zn deposited morphology and improving the corrosion resistance of the electrode by constructing an ideal interfacial is a typical strategy to achieve a long-cycle-life Zn metal anode [17–20]. Meanwhile, benefiting from the low surface energy and high atomic packing density of the Zn (002) crystal plane, Zn deposits exhibit compact and horizontally aligned crystallographic features that effectively inhibit dendrite growth and delay side reactions [21–23]. Therefore, the realization of Zn deposition with preferred (002) orientation by interface engineering offers exciting opportunities for enhancing the ability of the Zn anode to undergo long-term reversible plating/stripping [24–26]. However, owning to the surface heterogeneity of commercial Zn metal, Zn deposition with a non-planar structure can still be observed on the Zn anode surface, particularly at the grain boundaries and surface defects [27, 28]. Once this non-planar deposition morphology appears, it disrupts the uniform deposition order on the initial electrode surface [29], thereby limiting the plating/stripping ability of the Zn anode especially at high current densities [30].

Generally, the intrinsic condition for achieving dense metal deposition requires a current density lower than the classical diffusion-limited value (over-limited current density) [22, 31–33], which hinders the study of high-rate metal anodes. Manipulating the microstructure of the electrode to balance the interfacial electric field, redistribute the ion flux, and confine the metal growth region, thereby improving the electrochemical performance of metal electrodes [34–37], has been encouraged in the field of metal electrodeposition. Several studies have confirmed that the over-limiting current can be improved through the introduction of a negative surface charge in the microchannel interface, which controls the metal deposition behavior at a high current density [38–40]. Therefore, patterning the electrode surface and constructing an ingenious micro-interface structure are expected to realize a high-rate and long-cycle-life Zn metal anode.

**Fig. 1:**
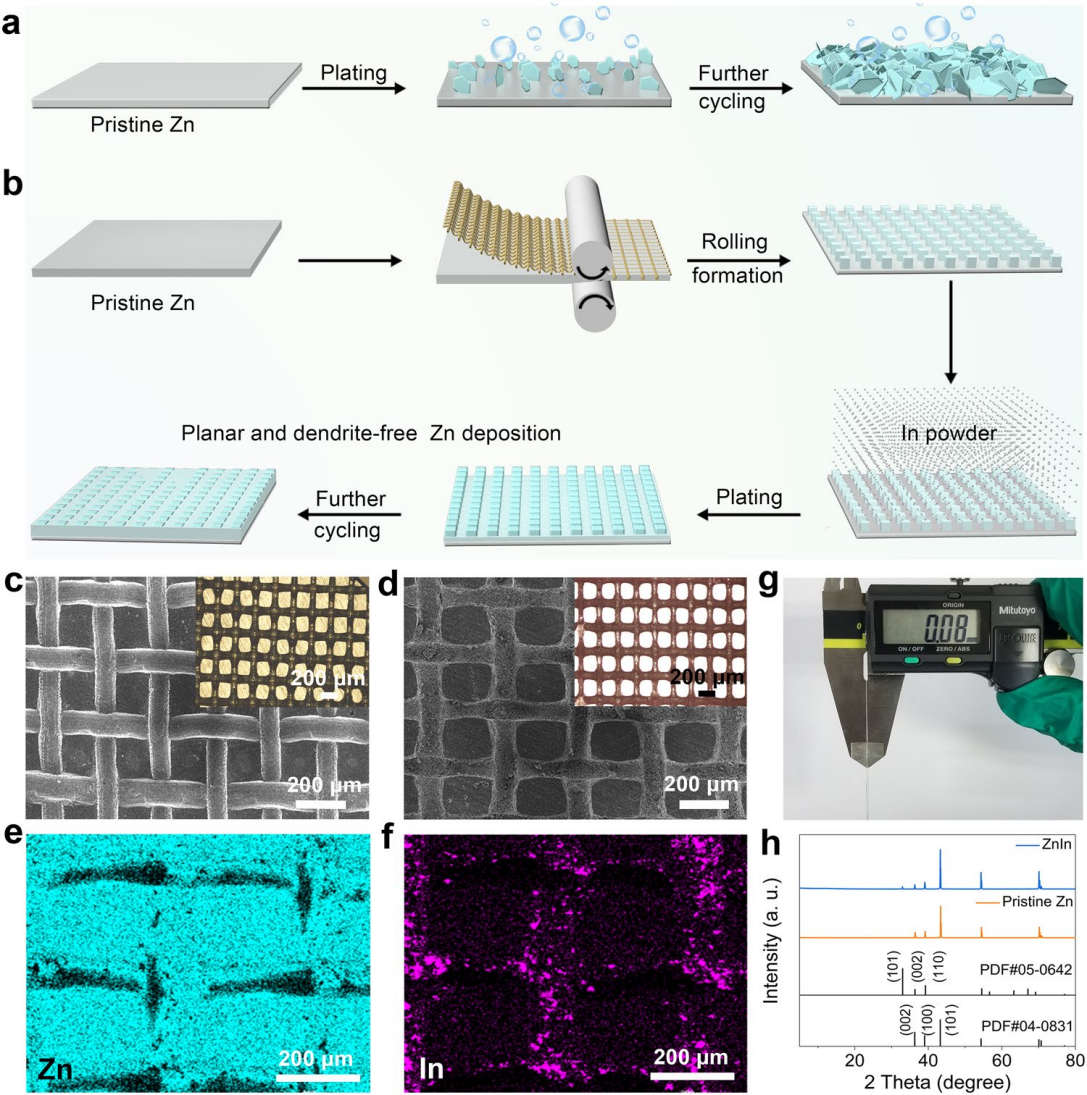
Fabrication and characterization of the ZnIn electrode. **a** Schematic illustration of Zn deposition morphology evolution on the pristine Zn anode and **b** preparation of the patterned ZnIn anode and the Zn plating behavior. SEM images of the **c** patterned Zn electrode and **d** ZnIn electrode (the inset figures show the corresponding optical microscopic images). **e, f** EDS elemental mapping images of the ZnIn electrode. **g** Thickness measurement of the prepared ZnIn electrode. **h** XRD patterns of the pristine Zn and ZnIn electrodes

In this work, we propose a Zn electrode surface patterning and endow a zincphilic Zn-Indium (In) interface layer in the microchannels to achieve the in-region preferential deposition of Zn ions, thereby achieving a high-rate and long-cycle-life Zn metal anode (ZnIn). Although research based on the chemical substitution or electrodeposition methods has confirmed that the In-based interface layer can effectively inhibit HER and improve the cycle life of the electrode [41–46], factors such as solution concentration, reaction time, environment temperature, and additive selection will affect the quality of the interface layer. Different from the above strategies, we prepared a patterned Zn electrode and selectively filled the microchannels with In powder to obtain a micro-interface architecture. Thanks to the higher reduction potential of In in the aqueous electrolyte, electrons on the micro-interface of the Zn electrode are accumulated on In, thus giving the electrode a high over-limiting current. In contrast to the inhomogeneous morphology of the pristine Zn (Fig. 1a), Zn ions on the ZnIn electrode can preferentially deposit heteroepitaxially at the Zn-In interface, finally achieving planar dendrite-free Zn deposition after the electrode surface tends to flatten with an increase in deposition capacity (Fig. 1b). The accumulation of electrons and zincophilicity in the microchannels improves the tolerance of the electrode at a high current density, enabling a long cycling life in ZnIn symmetric cells (over 25,000 cycles at the current densities of 10 and 20 mA cm^−2^). Furthermore, the as-prepared ZnIn anode coupled with iodine (I_2_)-anchored polyacrylonitrile-derived flexible carbon fibers (I_2_-CFs) cathode delivers a stable capacity over numerous cycles. The proposed strategy, which integrates patterned pretreatment and an ingenious interface architecture for metallic Zn electrodes, is expected to offer a rational design for the development of highly reversible ZMBs.

## Experimental Section

### Preparation of ZnIn Electrode

Commercial Zn foil (0.1 mm in thickness) and stainless steel mesh (100 mesh) were sonicated in acetone and ethanol, respectively, to clean the surface. The stainless steel mesh was covered with a Zn foil and passed through the rolling equipment. By continuously reducing the distance between the rollers, the patterned Zn (P-Zn) foil with a thickness of approximately 0.08 mm could be obtained after 4–6 times of the rolling process. The In powder was evenly filled on the microchannels of the P-Zn foil, and then the extra In powder on the electrode surface was wiped off with a cotton cloth, thereby enabling the In powder to remain in the microchannels rather than on the array surfaces. Subsequently, the prepared In-modified Zn foil was transferred to a tube furnace and kept at 180 °C with a rate of 2 °C min^−1^ for 2 h under an argon (Ar) atmosphere. Finally, the ZnIn electrode was obtained by a simple polishing and ultrasonic cleaning.

### Preparation of CFs

C_4_H_6_O_4_Zn·2H_2_O (0.5 g) was added into 10 mL of the dimethylformamide solution. Subsequently, polyacrylonitrile (1.0 g) (*M*_w_ = 150,000) was slowly added to the above solution and stirred at 40 °C for overnight. The obtained uniform and luminous yellow uniform solution was electrospun at a voltage of 15 kV and feeding rate of 1 mL h^−1^. The drum collector was set to 500 rpm at a distance of 15 cm from the needle. The as-prepared film was pre-oxidized at 240 °C for 2 h and then carbonized at 700 °C with a rate of 2 °C min^−1^ for 6 h under an Ar atmosphere to obtain the ZnO@CFs. Then, the ZnO@CFs was then soaked in a 1.0 M HCl solution and stirred for 2 h to remove the ZnO. Finally, the flexible porous CFs were obtained by washing with deionized water and ethanol, and drying at 60 °C in a vacuum oven for 6 h.

### Preparation of I_2_-CFs Cathode

I_2_ was uniformly dispersed in ethanol to prepare a 20 mg mL^−1^ solution. The prepared CFs were punched into a circular disk with a diameter of 12 mm and soaked in the I_2_ solution for 2 h. The Zn/I_2_ cell was assembled after the electrode dried naturally.

### Materials Characterization

The crystal structures were obtained by X-ray diffraction (XRD, D8 Advance (Bruker)) with monochromatic Cu K*a*. The morphologies of the electrodes were examined by using a field-emission scanning electron microscope (FE-SEM, MERLIN (Carl Zeiss)) and an optical microscope (Nikon, ECLIPSE LV 150N). The orientation of the plated crystal was determined using electron backscatter diffraction (FE-SEM, JEOL JSM-IT800). The electrode material was also observed by using a transmission electron microscope (TEM, FEI Talos F200i). The X-ray photoelectron spectroscopy (XPS) spectra were obtained using a K-alpha (Thermo Electron). The surface roughness of the Zn electrodes before and after cycling was detected by using a scanning probe microscope (SPM, Veeco D3100). The differential electrochemical mass spectrometry (DEMS) was carried out by the electrochemical mass spectrometry (HPR-20 EGA). The Zn symmetrical cells were purged and tested by Ar gas with a flow rate of 0.8 mL min^−1^.

### Electrochemical Measurement

Assembled coin cells (CR2023) for the Zn/Zn symmetric cells, Zn/Ti half cells, and Zn/I_2_ full cells were prepared in an open atmosphere. Glass fibers were used as the separator, and 2 M ZnSO_4_ was the electrolyte. For the plating/stripping, the charge/discharge, CE, and d*Q*/d*V* of the cells were carried out on WonaTech Automatic cell test instrument (WBCS3000). The capacitance–voltage (CV) curve, linear polarization, current vs. applied voltage (CA) curve, linear sweep voltammetry (LSV), and electrochemical impedance spectroscopy (EIS) measurements were performed using an Ivium Stat electrochemical workstation. The CV curves of the Zn plating/stripping were measured at a scan rate of 0.1 mV s^−1^ in a symmetric cell. Linear polarization was recorded at 5 mV s^−1^. The CA curves of the symmetrical Zn/Zn symmetric cells were obtained at a constant potential of -150 mV. The hydrogen evolution curves were tested by LSV in a 1 M NaSO_4_ electrolyte at a scan rate of 5 mV s^−1^ in a three-electrode configuration. The EIS curves were tested on the electrochemical workstation with a frequency range of 0.01–100 kHz. The Zn/I_2_ cells were cycled at 0.6 and 1.6 V.

### Computational Simulation

A simplified 3D model based on the COMSOL Multiphasic software was performed to simulate the current density and the Zn-ion concentration to investigate the dynamic deposition mechanism of the Zn ions. The size of each Zn embossing module was 0.2 × 0.2 mm^2^, and they were spaced 0.1 mm apart. The current density on the Zn electrode was calculated using the Butler–Volmer equation:

1 $$i_{{{\text{loc}}}} = i_{0} \left( {{\text{exp}}\left( {\frac{{\alpha_{a} F\eta }}{{{\text{RT}}}}} \right) - {\text{exp}}\left( {\frac{{ - \alpha_{c} F\eta }}{{{\text{RT}}}}} \right)} \right)$$

where *i*_loc_ represents the current density on the electrode surface, *i*_*0*_ is the exchange current density, *η* represents the activation overpotential, and *α*_*a*_ and *α*_*c*_ are the charge transfer coefficients in the anode directions, respectively. *R* and *T* are the ideal gas constant and the temperature of 298 K, respectively.

The Zn-ion concentration distribution needs to consider the ionic migration and diffusion under an established electric field, which follows the Ness–Einstein equation:

2 $$N_{i} = - D_{i} \nabla c_{i} - z_{i} u_{m,i} Fc_{i} \nabla \phi_{l} + uc_{i}$$

where *N*_*i*_ is the Zn-ion flux, *D*_*i*_ is the diffusion coefficient, *Z*_*i*_ represents the Zn-ion transfer number, and *u*_*m,i*_ is the electric mobility coefficient. F and* ϕ* represent the Faraday constant and potential, respectively.

### DFT Calculations

First-principles calculations based on density functional theory (DFT) were performed using the generalized gradient approximation (GGA) and the Perdew–Burke–Ernzerhof (PBE) exchange–correlation functional. 3 × 3 Zn slabs with four atomic layers were built for both the Zn (002) and Zn (100) surfaces, and 2 × 3 In slabs with four atomic layers were constructed for both the In (002) and In (110) surfaces. The vacuum layer thickness of 15 Å was employed. The self-consistent calculations apply a convergence energy threshold of 0.5 × 10^–6^ eV. The cutoff energy was set to 600 eV. The k-points were chosen by the Monkhorst–Pack method. For the structural convergence criterion, energy, force, stress, and displacement are set to 1.0 × 10^-5^ eV atom^−1^, 0.03 eV Å^−1^, 0.05 GPa, and 0.001 Å, respectively. The binding energies were calculated using Eq. (3):

3 $$E_{{{\text{ads}}}} = E_{{{\text{stotal}}}} - E_{{{\text{base}}}} - E_{{{\text{Zn}}}}$$

The free energy of the hydrogen adsorption at equilibrium was calculated by Eq. (4):

4 $$\Delta G_{H} = \Delta E_{H} + \Delta E_{{{\text{ZPE}}}} - T\Delta S$$

with

5 $$\Delta E_{H} = \Delta E_{{\left( {{\text{surface}} + H} \right)}} - \Delta E_{{{\text{surface}}}} - \frac{1}{2}E_{{H_{2} }}$$

6 $$\Delta {\text{ZPE}} = {\text{ZPE}}_{H} - \frac{1}{2}{\text{ZPE}}_{{H_{2} }}$$

7 $${ }T\Delta S = \frac{1}{2}{\text{ZPS}}_{{H_{2} }} = - 0.205$$

where $\Delta E_{{\left( {{\text{surface}} + H} \right)}}$ is the total energy of the H atom absorbed on the different planes, and $\Delta E_{{{\text{surface}}}}$ and $E_{{H_{2} }}$ refer the energies of the pristine system and the H_2_ molecule, respectively. $\Delta$ ZPE represents the gap of the zero-point energy of the H-atom and the H_2_ molecule.

## Results and Discussion

### Fabrication and Characterization of ZnIn Electrode

Considering the moderate ductility and softness of metallic Zn at room temperature, this facilitates the design of Zn metal surface microstructure [47]. In detail, Zn foil surface was covered with a stainless steel mesh (Fig. S1a, b), and the mesh was removed after continuous rolling to obtain a Zn foil with square arrays and microchannels). Subsequently, the In powder (Fig. S1c, d) was selectively filled in the interconnected microchannels to prepare the ZnIn electrode with a zincophilic Zn-In interface after a simple annealing approach. Figure 1c shows a typical SEM image of the P-Zn electrode. The optical microscope photograph reveals that the neatly arranged Zn arrays have a size of approximately 200 μm, and the intervals are spaced about 100 μm apart (Fig. S2a). The microstructure of the ZnIn electrode is shown in Figs. 1d and S2b, which reveals that the array surface is smooth, while the microchannels are rough, implying that the In powder is anchored in the microchannels. In addition, the corresponding energy-dispersive X-ray spectroscopy (EDS) mappings reflect the assigned positions of Zn and In, indicating that Zn and In are mainly distributed on the array surface (Fig. 1e) and in the microchannels (Fig. 1f), respectively. A digital photograph displays the ZnIn electrode with a thickness of approximately 80 μm fabricated by continuously adjusting the height during the rolling process (Fig. 1g). By comparing the patterned Zn electrodes before and after In powder modification, we calculated the mass proportion of In on the ZnIn electrode (4.0 cm × 7.0 cm). Figure S3 shows the quality of the Zn foil after patterning. Compared with the ZnIn electrode modified by In powder, it can be found that the mass loading per unit area of In powder is about 1.0 mg cm^−2^. From the XRD pattern of Fig. 1h, it is evident that the ZnIn electrode has an additional diffraction peak at 2*θ* = 32.9° compared to the pristine Zn, which corresponds to the (101) plane of In (PDF#05–0642) [46].

### Morphologies Evolution of Zn Deposition on the ZnIn Electrode

**Fig. 2:**
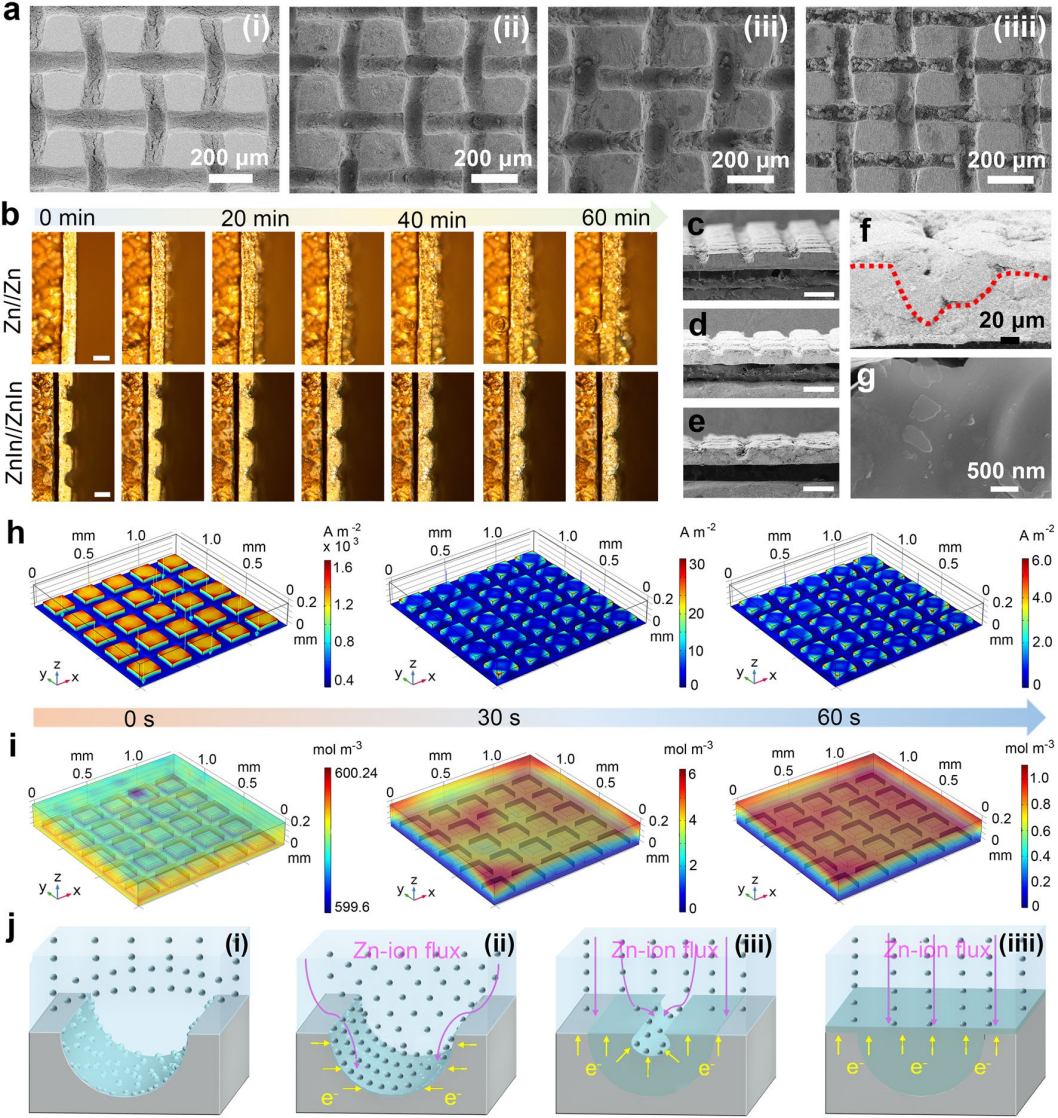
Dynamic evolution of the ZnIn electrode morphology during Zn plating and analysis of the underlying mechanism. **a** SEM images of the ZnIn electrode after Zn deposition of (i) 0 mAh cm^−2^, (ii) 3.0 mAh cm^−2^, (iii) 5.0 mAh cm^−2^, and (iv) 10.0 mAh cm^−2^. **b** In situ optical observations of Zn plating behavior of pristine Zn and ZnIn electrodes. Scale bars are 100 μm. Cross-sectional SEM images for the Zn deposition with different areal capacities of **c** 5.0 mAh cm^−2^, **d** 10.0 mAh cm^−2^, and **e** 15.0 mAh cm^−2^ (Scale bars are 200 μm) and **f** magnified image of **e**. **g** SEM image of the planar deposited Zn flakes. Simulation of the evolution process of **h** current density distribution and **i** Zn-ion concentration distribution on the ZnIn electrode surface at different times. **j** Schematic illustration of the morphology evolution of the ZnIn electrode during Zn deposition

To investigate the effect of the ZnIn electrode on the Zn metal deposition behavior, the morphological evolution with various deposition capacities was characterized using SEM and optical microscope images. Comparing the pristine ZnIn electrode (Figs. 2ai and S4ai), it can be observed that the deposited Zn is predominantly accumulated in the microchannels and grows with the capacity increasing from 3.0 to 10.0 mAh cm^−2^ (Figs. 2aii–iv and S4b–d). Meanwhile, more direct visual monitoring of the Zn metal plating process can be obtained by the in situ optical microscopy. For the pristine Zn, a heterogeneous Zn deposition morphology is observed after plating for 10 min with an applied constant current density of 10 mA cm^−2^. As the deposition time increases, the protrusions on the electrode surface gradually evolve into Zn dendrites (Fig. 2b and Video S1). In contrast, almost no deposits are observed on the ZnIn electrode array surface within 30 min, while bright deposits can be found in the microchannels and increase with time, demonstrating the intraregional selective preferential deposition of Zn metal. After performing 40 min deposition, the newly deposited Zn metal almost filled the microchannel. Subsequently, Zn ions are deposited on the array surface and display a planar deposition morphology (Fig. 2b and Video S2). Figure S5 and Video S3 show the Zn deposition on the P-Zn. Similar to the pristine Zn electrode, protrusions appear on the surface of the array after performing 10 min plating, and eventually evolve into the notorious dendrites. In contrast to the ZnIn electrode, the newly deposited metal is not preferentially deposited in the microchannels of the P-Zn electrode, confirming that the ZnIn interface can induce selective preferential Zn deposition.

Figure 2c–e shows the cross-sectional SEM images of the ZnIn electrodes with the capacitiy of 5.0, 10.0, and 15.0 mAh cm^−2^, respectively. The thickness of the ZnIn electrodes does not vary at the capacities of 5.0 and 10.0 mAh cm^−2^, implying that the Zn atoms are selectively preferentially plated in microchannels. The surface morphology of the Zn plating capacity at 15.0 mAh cm^−2^ (Fig. S6) shows dense Zn deposits on the array surface, which indicates that the selective deposition of Zn atoms in the microchannel region changes to uniform deposition on the electrode surface. Additionally, the partially magnified SEM image in Fig. 2e clearly and representatively shows that the newly deposited Zn metal fills the entire microchannel and exhibits a smooth surface (Fig. 2f). The horizontally arranged Zn flakes with a size of approximately 500 nm in Fig. 2g indicate that the ZnIn electrode has the ability to guide the oriented growth of the Zn (002) plane [9, 48]. However, Fig. S7 shows the vertical arrangement of Zn flakes on the pristine Zn substrate, which is not conducive to uniform Zn deposition and induces the Zn dendrite growth [24, 25].

### Zn Deposition on the ZnIn Electrode

The surface chemistry of the pristine ZnIn and the ZnIn electrodes after plating of 3.0 mAh cm^−2^ was characterized by XPS. The XPS full survey scan spectra of the two electrodes reveal the signals of Zn 2*p* and In 3*d* peaks (Fig. S8a). The high-resolution core-level Zn 2*p* spectrum displays small shift toward the lower binding energy when plated to 3.0 mAh cm^−2^, which implies that the electrons of the interfacial are more inclined to accumulate on In (Fig. S8b) [49, 50]. Besides, the spectrum of In 3*d*_5/2_ (Fig. S8c) reveals two peaks at 444.5 and 445.6 eV, which can be attributed the In-In (II) and In-O (I) bonds, respectively [51]. The increase in the proportion of the In-O (I) band after Zn deposition suggests that the In at the Zn-In interface layer combines with dissolved oxygen more readily than Zn, facilitating more efficient Zn plating/stripping [52]. Furthermore, the wettability of the electrode surface can roughly feedback the distribution of Zn ions at the electrode/electrolyte interface. This is because good wettability can effectively improve the uniform distribution of Zn ions at the interface, increase the free energy of the electrode/electrolyte interface, and promote the Zn-ion transmission kinetics on the electrode surface [53, 54]. Figure S9 depicts the contact angles of the 2 M ZnSO_4_ electrolyte on the pristine Zn, P-Zn, and ZnIn electrodes, respectively. The larger contact angle (112.9°) of the ZnIn electrode compared with that of the pristine Zn (71.2°) implies the poor wetting of the ZnIn electrode (Fig. S9a). In addition, the P-Zn electrode also shows a poor wettability, even with a Zn deposition capacity of 3.0 mAh cm^−2^, which may be attributed to the non-uniform Zn deposition on the P-Zn electrode surface. (Fig. S9b, c) In contrast, the contact angle of the ZnIn electrode decreases with increasing deposition capacity (Fig. S9d-f), which indicates that the new deposits improved the wettability of the electrode and thus redistributed the Zn-ion current on the electrode surface.

To further explore the Zn plating process on the electrodes, the current density and Zn-ion concentration distribution on the electrode surface were simulated using COMSOL Multiphysics software. As shown in Fig. S10a, the current density on the pristine Zn electrode displays an inhomogeneous distribution, with the maximum current concentration at the tip of the protrusion. Combined with the simulation of the Zn-ion concentration distribution, it determines that the protrusion position has a higher concentration (Fig. S10b). The large current density and high Zn-ion concentration at the tip sites accelerate the uneven deposition of Zn metal on these components, resulting in the dendrite growth. The dynamic current density and Zn-ion concentration distribution simulations of the ZnIn electrode are shown in Figs. 2h, i and S11. In the initial state, an ultrahigh current density accumulation is observed on the array surface, which is attributable to the height difference between the array surface and the microchannel. Notably, the current density on the array surface decreases continuously with the Zn plating time, while it increases in the microchannel (Fig. S12), possibly owing the over-limiting current in the microchannels [38]. This phenomenon suggests that the Zn ions are preferentially deposited in the microchannel during the early stage of plating, consistent with the conclusions from ex situ SEM and in situ optical microscope analyses. When Zn plating was performed for 30 s, the current density on the electrode surface decreased by ~ 50 times compared to that in the initial state, implying that the current density on the electrode surface tended to be uniform. In addition, the surface current density becomes even in the final state, indicating the subsequent planar Zn deposition. Figure S13 shows that the Zn-ion concentration in the microchannels gradually decreases with the continuation of plating and finally tends to be homogeneous. This suggests that the electron gain of the Zn ions mainly occurs in the Zn-In interface microchannels during the initial plating/stripping process, further illustrating that the Zn ions are preferentially deposited in the microchannels. With an increase in the plating time, the electrode tends to be flat after the microchannel is filled with the newly Zn deposit, and the Zn-ion flux on the electrode surface becomes uniform, thus leading to the planar Zn deposition.

**Fig. 3:**
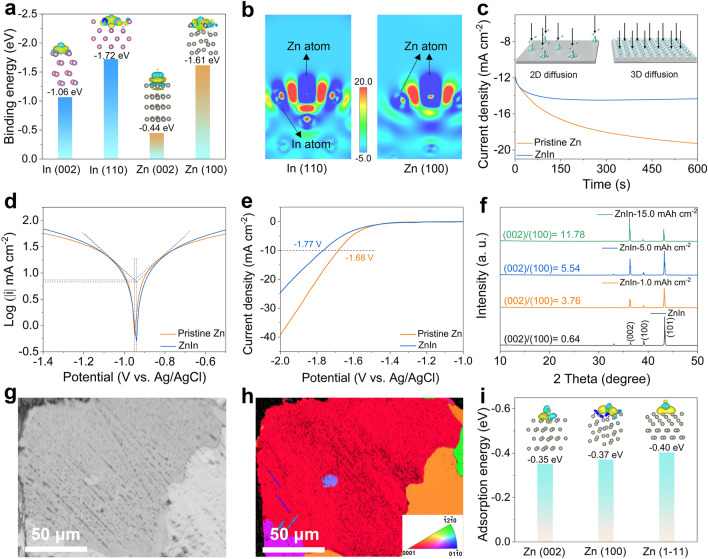
Side reaction resistance of the ZnIn electrode. **a** Binding energy barrier for Zn atom on In (002), In (110), Zn (002), and Zn (100) planes (the insets show the corresponding charge density difference, respectively) and **b** corresponding sliced 2D contour map of the Zn atom bonded with In (110) and Zn (100) planes. **c** Chronoamperometry curves, **d** linear polarization curves, and **e** hydrogen evolution polarization curves of the pristine Zn and ZnIn electrodes. **f** XRD patterns of the ZnIn electrodes with different area capacities. **g** Band contrast image and **h** corresponding EBSD mapping image of the ZnIn electrode after cycling 100 h. **i** Absorption energy of H_2_O molecule on various Zn crystal planes (the insets show the corresponding charge density difference, respectively)

Therefore, a simplified diagram of Zn deposition on the ZnIn electrode is depicted in Fig. 2j. During the Zn deposition, electrons tend to accumulate on the In surface with a higher reduction potential [44]. This enhances the transport of Zn ions in the microchannel, thus realizing the heteroepitaxial Zn deposition at the Zn-In interface. With an increase in the deposition capacity, the electrode tends to be flat, and the Zn-ion flux is redistributed, thereby achieving dendrite-free Zn deposition. DFT calculations were used to gain further insight into the binding energies between Zn atoms and different Zn and In crystal planes. As shown in Fig. 3a, the In (110) plane depicts a higher binding energy than the Zn (100) and Zn (002) planes, suggesting that the metallic In can lower the Zn nucleation barrier and induce selective Zn deposition. The slices of the electron density difference map (Figs. 3b and S14) show an electron-rich environment on the In atom surface, which indicates that the formed Zn-In interface has a high affinity for the Zn atom and confirms that the Zn is preferentially deposited in the microchannels. Consequently, the Zn-ion diffusion mechanism changes from the two-dimensional (2D) diffusion on the pristine Zn electrode surface to the 3D diffusion on the ZnIn electrode (Fig. 3c) [55]. Meanwhile, benefiting from the ingenious structural design and the zincophilicity of the Zn-In interface, the ZnIn electrode displays higher Zn-ion transfer number (0.40) than the pristine Zn electrode (0.29) in Zn symmetric cells (Fig. S15). In addition, the potential of the Zn plating on the ZnIn electrode is higher than that on pristine Zn electrode (|AB|= 35 mV), implying smaller Zn nucleus and more nucleation sites on the ZnIn electrode (Fig. S16), which is favorable for dense and uniform Zn metal deposition. The 3D Zn-ion diffusion mechanism and high Zn-ion transport number indicate a uniform surface electric field and Zn-ion concentration gradient on the ZnIn electrode, which favors the formation of dense and smooth Zn deposits [56, 57]. Moreover, hydrated Zn ions are deposited on the surface of the Zn electrode after undergoing a desolvation process, and there is charge migration at the interface. Typically, the desolvation energy barrier can be described by the activation energy (*E*_*a*_), which follows the Arrhenius equation [47]:${ }1/R_{ct} = {\text{Aexp}}\left( { - E_{a} /{\text{RT}}} \right)$, where *R*_ct_ and A represent the interfacial resistance and frequency factor, and R and *T* are the gas constant and absolute temperature, respectively. As shown in Fig. S17, the *R*_*ct*_ of the ZnIn electrode is smaller than that of pristine Zn at different temperatures, implying faster charge transfer kinetics in the ZnIn symmetric cell. Therefore, the ZnIn electrode depicts a lower *E*_a_ (12.9 kJ mol^−1^) than the pristine Zn electrode (16.4 kJ mol^−1^), which implies that the ingenious ZnIn micro-interface layer design can reduce the activation energy of Zn deposition, thus enhance the transfer kinetics of Zn ions.

### Side Reaction Resistance and Zn Deposition Mechanism

Linear polarization was carried out to study the corrosion resistance of the pristine Zn and ZnIn electrodes (Fig. 3d). The corrosion potential of the ZnIn electrode (− 0.94 V) is higher than that of the pristine Zn electrode (− 0.95 V). Correspondingly, the ZnIn electrode lowers the corrosion current by 390 μA cm^−2^, indicating that the ZnIn electrode can inhibit the side reaction and enhance the corrosion resistance. Meanwhile, LSV was employed to evaluate the HER of different Zn electrodes. As presented in Fig. 3e, the ZnIn electrode has a larger overpotential than the pristine Zn electrode at 10.0 mA cm^−2^, which implies that the ZnIn electrode is more potent in suppressing HER than the Zn electrode [58, 59]. The XRD patterns of the ZnIn electrodes with different areal capacities reflect the crystallographic orientation of the Zn deposits on the ZnIn electrodes (Fig. 3f). The value of *I*_*(002)*_*/I*_*(100)*_ increases with the deposition capacity, implying that more (002) planes are exposed, which suggests that the Zn-In interface can achieve homogeneous and planar Zn deposition. Additionally, the optical microscopic images reveal the surface morphologies of the pristine Zn after cycling for 100 h at 1.0 mA cm^−2^ and 1.0 mAh cm^−2^. Several large and rough Zn protrusions can be observed on the electrode surface (Fig. S18a), which will trigger the “tip effect” and lead to the formation of dendrites during the subsequent Zn deposition [29, 31]. Similarly, the P-Zn electrode array surface exhibits protrusions and dendrites (Fig. S18b), indicating non-uniform Zn deposition. In sharp contrast, the array surface of the ZnIn electrode exhibits a smooth and flat morphology (Fig. S18c). Some parallel-arranged Zn flakes with a size of ~ 50 μm are found on the array surface (Fig. S18d), corresponding to the close-packed (002) plane and suggesting corrosion-resistant and dendrite-free stripping/plating behavior [27, 60]. Additionally, electron backscatter diffraction (EBSD) was employed to further confirm the crystal plane orientation of the ZnIn electrode before and after cycling. The band contrast image and corresponding 2D color-coded map of the ZnIn electrode before cycling reveal multiple grains and a differently oriented surface (Fig. S19a, b), indicating its heterogeneous crystallographic orientation. However, the ZnIn electrode after performing 100 h of plating/stripping shows large grains and primarily red series-dominance (Figs. 3g, h, and S19c, d), corresponding to the Zn ions along the (002) plane of deposition. Furthermore, DFT calculations show that the Zn (002) plane has a lower binding energy to water molecule than other Zn crystal planes (Fig. 3i), confirming the excellent anticorrosion properties of the ZnIn electrode with the (002)-orientated Zn deposition.

**Fig. 4:**
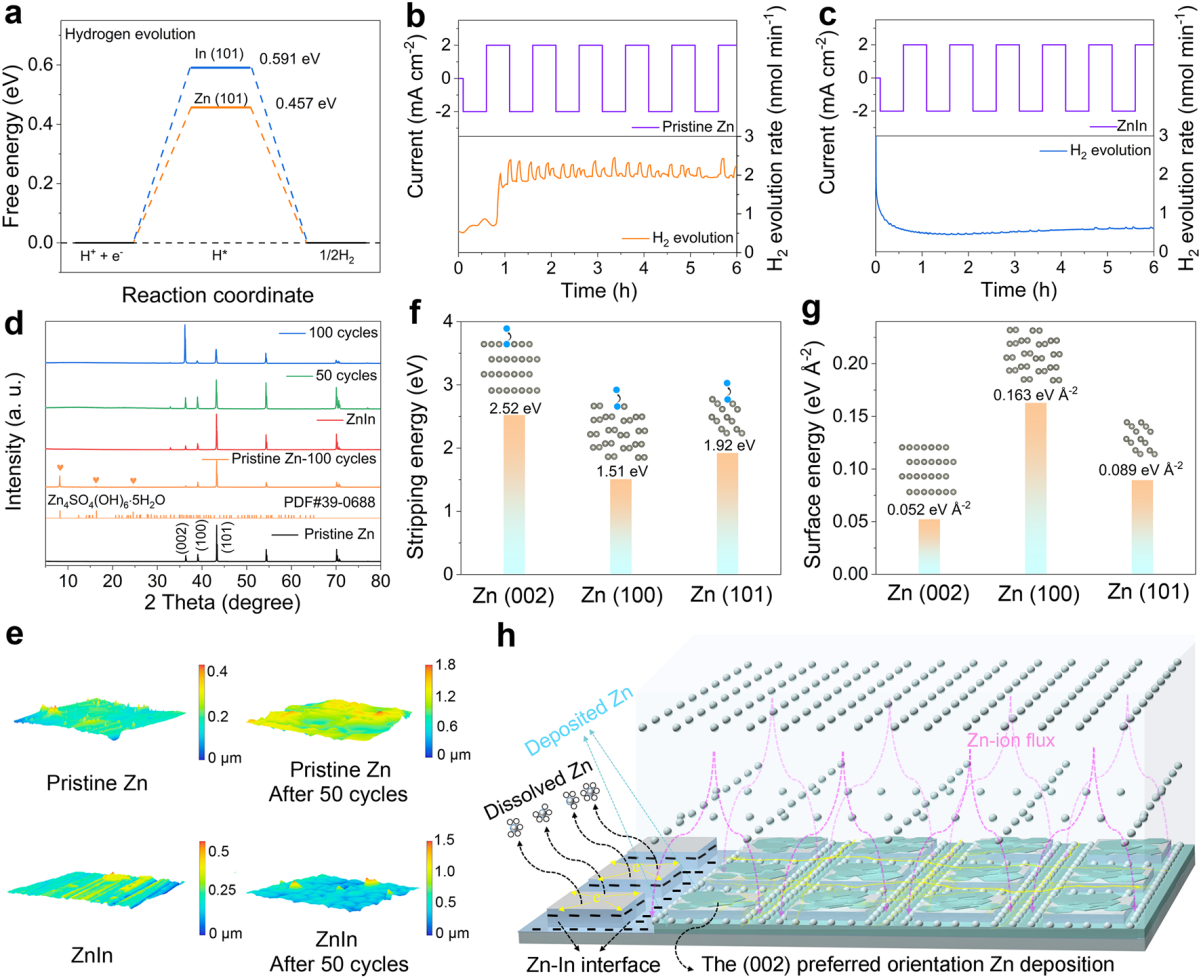
Zn deposition mechanism of the ZnIn electrode. **a** Free energy diagram for the HER. DEMS plots of H_2_ evolution rate of the **b** pristine Zn and **c** ZnIn symmetrical cells during the continuous plating, respectively. **d** XRD patterns and **e** surface roughness of the different electrodes before and after cycling test. The current density is 2.0 mA cm^−2^. **f** Stripping energy of Zn atom from different crystal planes and **g** surface energy of the different Zn planes. **h** Schematic depiction of Zn deposition behavior on the ZnIn electrode

As shown in Fig. 4a, the free energy of H adsorption on the In (101) is 0.591 eV, which is higher than that of the Zn (101) (0.457 eV), indicating that the HER can be inhibited by introducing the micro-interface. The DEMS was carried out to monitor the hydrogen generation with the cycle time for the different electrodes in symmetrical cells. For the pristine Zn symmetrical cell (Fig. 4b), the H_2_ concentration increases fast with the plating/stripping time, attributing to the severe side reaction between the Zn electrode and electrolyte. In sharp contrast, the produced H_2_ with a lower concentration is detected in the ZnIn symmetrical cell during the continuous plating/stripping process of 6 h (Fig. 4c), implying that the ZnIn electrode has excellent anticorrosion ability. To further illustrate the inhibition ability of the ZnIn electrode on HER, the surface by-products of the pristine Zn and ZnIn electrodes before and after cycling were analyzed by the XRD (Fig. 4d). The Zn_4_SO_4_(OH)_6_·5H_2_O peaks caused by the hydrogen evolution can be detected on the pristine Zn electrode after 50 cycles. In contrast, no by-product peaks are detected on the ZnIn electrode after 50 cycles and display a preferential (002) direction Zn deposition as the cycle number increases, suggesting the excellent corrosion resistance of the ZnIn electrode. The surface roughness of the different electrodes after 50 cycles at the current density of 1.0 mA cm^−2^ is shown in Fig. 4e. The cycled ZnIn electrode displays a smoother surface than the pristine Zn electrode, attributing to the homoepitaxial Zn metal deposition on the ZnIn array surface and reducing the risk of dendrite formation. Additionally, Fig. S20 reflects the Mulliken charge distributions of the Zn/In and Zn/Zn interfaces. The In (002) and In (110) crystal planes show a more negative surface charge distribution than the Zn (002) and Zn (100), which indicates that electrons are distributed near the In side. For the as-prepared ZnIn electrode, the electrons tend to accumulate on the In side in the microchannel, which means that In powder into the electrode microchannel is beneficial to the migration of electrons from the Zn electrode surface. The Zn atoms close to In are more easily to lose electrons, thereby showing faster Zn dissolution during the Zn stripping. However, the zincophilic In interface with high binding energy preferentially guides the Zn atoms to deposit in the microchannel. Additionally, DFT results show the stripping energy of the Zn atom from different Zn crystals (Fig. 4f). The energy consumption of the Zn atom is higher to be stripped from the Zn (002) (2.52 V) plane than that from the Zn (100) (1.51 eV) and Zn (101) (1.92 eV) planes, indicating that non-(002) planar Zn atoms are unstable and easy to dissolve, and more (002) crystal planes are exposed on the array surface. Meanwhile, Fig. 4g displays the surface energy of the different Zn planes, which shows that the Zn (002) plane with the lower surface energy of 0.052 eV Å^−2^ than those of the Zn (100) (0.163 eV Å^−2^) and Zn (101) (0.089 eV Å^−2^) planes. Therefore, the rapid stripping of Zn atoms on the non-(002) planes and the lower surface energy of the (002) plane facilitates the subsequent Zn deposition along the (002) direction. The mechanism of Zn deposition on the ZnIn electrodes can be systematically summarized as shown in Fig. 4h. The Zn electrode is patterned and endows a zincphilic Zn-In interface in the microchannel, where the obtained ZnIn electrode can guide Zn ions to be preferentially deposited in the microchannels and finally achieve the Zn ions to deposit along the (002) plane. In particular, the Zn-In interface constructed in the microchannel induces the electrode surface electrons to be more inclined to gather on the In side, which promotes a large number of the Zn ions to obtain electrons and realizes the heteroepitaxial deposition of the (002) orientation Zn in the microchannel. The non-(002) facet loosely packed Zn atoms on the surface of the array have high reactivity and are preferentially stripped. A small number of Zn ions on the array surface tends to be homoepitaxially deposited on the (002) plane to achieve a lower surface energy and are typically stable during the plating process. Therefore, the ZnIn electrode can obtain a (002) plane-oriented Zn deposition, thereby suppressing dendrite growth and side reactions.

### Electrochemical Performance of ZnIn Electrode

**Fig. 5:**
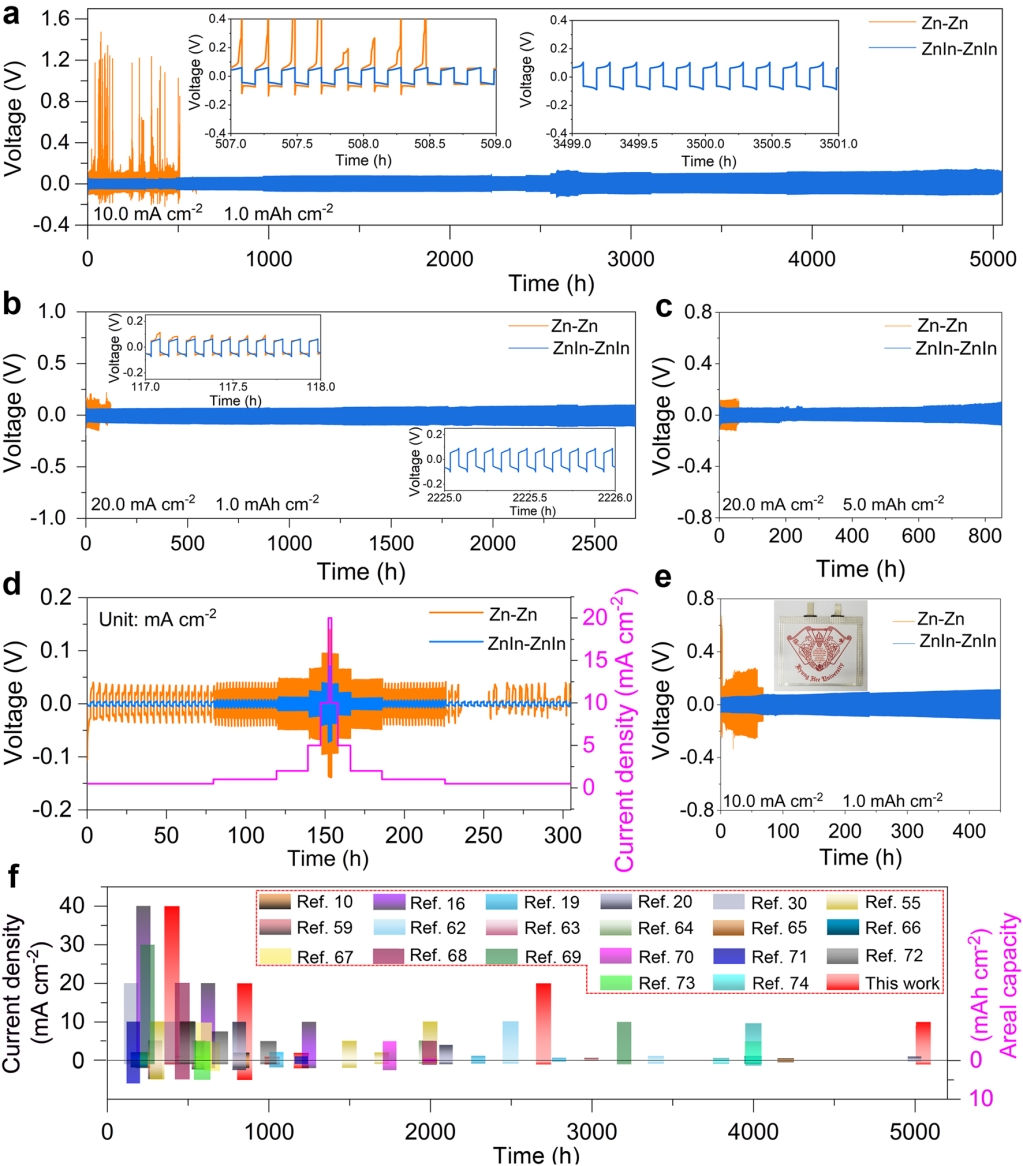
Electrochemical performance characterizations of Zn metal deposition on the difference electrodes. Galvanostatic cycling of symmetrical ZnIn and pristine Zn cells with the plating-stripping conditions of **a**10.0 mA cm^−2^ and 1.0 mAh cm^−2^, **b** 20.0 mA cm^−2^ and 1.0 mAh cm^−2^ (the insets show the magnified curves at specific cycles), and **c** 20.0 mA cm^−2^ and 5.0 mAh cm^−2^. **d** Rate performance of the ZnIn and pristine Zn symmetric cells. **e** Galvanostatic cycling of the ZnIn and pristine Zn symmetric pouch cells at 10.0 mA cm^−2^ and 1.0 mAh cm^−2^. **f** Comparative cycling performance of the ZnIn anode with others

The voltage profiles of the Zn deposition at various current densities are shown in Fig. S21. Notably, the ZnIn electrode exhibits nearly zero nucleation overpotential at a current density of 0.5 mA cm^−2^ (Fig. S21a). Meanwhile, when the current density increases to 1.0 and 2.0 mA cm^−2^, the nucleation overpotentials of the ZnIn electrode are 6.5 and 7.0 mV, respectively, which are still much lower than those of the pristine Zn electrode (Fig. S21b, c). This indicates that the Zn-In interface promotes Zn nucleation and significantly reduces the nucleation barrier of Zn plating [49]. The reversible Zn plating/stripping was evaluated by galvanostatic cycling in the symmetric cells. The ZnIn electrode depicts stable plating/stripping and a small voltage hysteresis for more than 1,100 h at 1.0 mA cm^−2^ with a capacity of 1.0 mAh cm^−2^. In contrast, for the P-Zn and pristine Zn symmetric cells, the voltage drops at 170 and 230 h, respectively, which is attributed to the Zn dendrite growth, resulting in the cell short circuit (Fig. S22a, b). At a current density of 2.0 mA cm^−2^, the ZnIn symmetrical cell can operate over 1,200 h and also deliver the lowest voltage hysteresis of 13.9 mV, which is significantly better than those of the P-Zn and pristine Zn symmetric cells (Fig. S22c, d). The small voltage hysteresis indicates that the ZnIn electrode can enhance the Zn-ion transfer kinetics, which is beneficial for the development of high-rate ZMBs [61]. Therefore, the Zn symmetric cell was operated at a current density of 10.0 mA cm^−2^ and a capacity of 1.0 mAh cm^−2^. As shown in Fig. 5a, the ZnIn symmetric cell shows an ultra-long-cycle life of more than 5,050 h (7 months, over 25,000 cycles), which indicates the outstanding electrochemical stability of the ZnIn electrode. In contrast, the cycle life of the P-Zn and pristine Zn symmetric cells are only about 510 and 660 h, respectively (Fig. S23). Additionally, the ZnIn/Ti half cells deliver an average CE value of 99.9% over 500 cycles at 1.0 mA cm^–2^ (Fig. S24a) and 99.4% over 1,000 cycles at 10.0 mA cm^–2^ (Fig. S24b). However, the fluctuating CE of Zn/Ti half cells can be obvious at 98 and 185 cycles, respectively. In addition, the ZnIn/Ti half cells reflect more stable voltage profile curves at various cycles compared to those of the Zn/Ti half cells at 10.0 mA cm^–2^ (Fig. S24c-e), demonstrating excellent electrochemical reversibility. Furthermore, the current–voltage curves (Fig. S25) show that the Zn symmetric cell exhibits a current of − 60.3 mA at − 0.21 V and remains stable at − 43.8 mA, while the ZnIn symmetric cell displays a higher current of − 101.7 mA at − 0.28 V, which suggests that the aggregation of electrons in the electrode microchannel enhances the over-limiting current of the ZnIn electrode and improves its tolerance of the electrode at high current density [39].

Therefore, the galvanostatic cycling of the Zn symmetric cells was performed at higher current densities to explore the high-rate performance of the ZnIn electrode. As shown in Fig. 5b, the ZnIn symmetric cell exhibits remarkable durability, operating smoothly for more than 2,700 h (27,000 cycles) at a current density of 20.0 mA cm^−2^ and a capacity of 1.0 mAh cm^−2^, while the Zn symmetric cell suffers short circuit at 117 h. Additionally, when the current density increases to 40.0 mA cm^−2^, the ZnIn symmetric cell can also deliver a stable plating/stripping for 400 h (Fig. S26), which implies that the over-limited current in the ZnIn electrode improves the stability of Zn deposition at high current density. In addition, the large-area capacity measurements were performed to further evaluate the advancement of the prepared ZnIn electrodes. As shown in Figs. 5c and S27, the ZnIn electrode delivers a stable plating/stripping life of up to 850 h at a current density of 20.0 mA cm^−2^ with a capacity of 5.0 mAh cm^−2^ outperforming to the pristine Zn symmetric cell with a plating life of 60 h. Furthermore, the ZnIn symmetric cell exhibits stable operation for 500 h at 2.0 mA cm^−2^ and 10.0 mAh cm^−2^ (Fig. S28), and about 200 h at 10.0 mA cm^−2^ and 10.0 mAh cm^−2^ (Fig. S29). Figure 5d shows the rate performances of the Zn symmetric cells at various current densities from 0.5 to 20.0 mA cm^−2^. A slight change of the overpotential in the ZnIn electrode at 150 h may be attributed to the designed ZnIn electrode, increasing the over-limiting current of the electrode. Therefore, under high-rate working conditions, the over-limiting current improves the tolerance of the electrode, thereby showing a reduction in overpotential and subsequent stable plating/stripping during the cycling process. The lower voltage hysteresis, longer cycle life, and higher cumulative capacity of the ZnIn electrode indicate the excellent stability of the ZnIn symmetric cell (Fig. S30a, b). Furthermore, as shown in Fig. 5e, the ZnIn symmetric pouch cell delivers an excellent cycling performance for more than 450 h at 10.0 mA cm^−2^ (40.0 mA) and 1.0 mAh cm^−2^ (4.0 mAh). As a result, the ZnIn electrode outperformed most of the previously reported electrodes in terms of overall electrochemical performance (Fig. 5f and Table S1) [10, 16, 19, 20, 30, 55, 59, 62–74].

### Electrochemical Performance of Aqueous Zn/I_2_ Cells

**Fig. 6:**
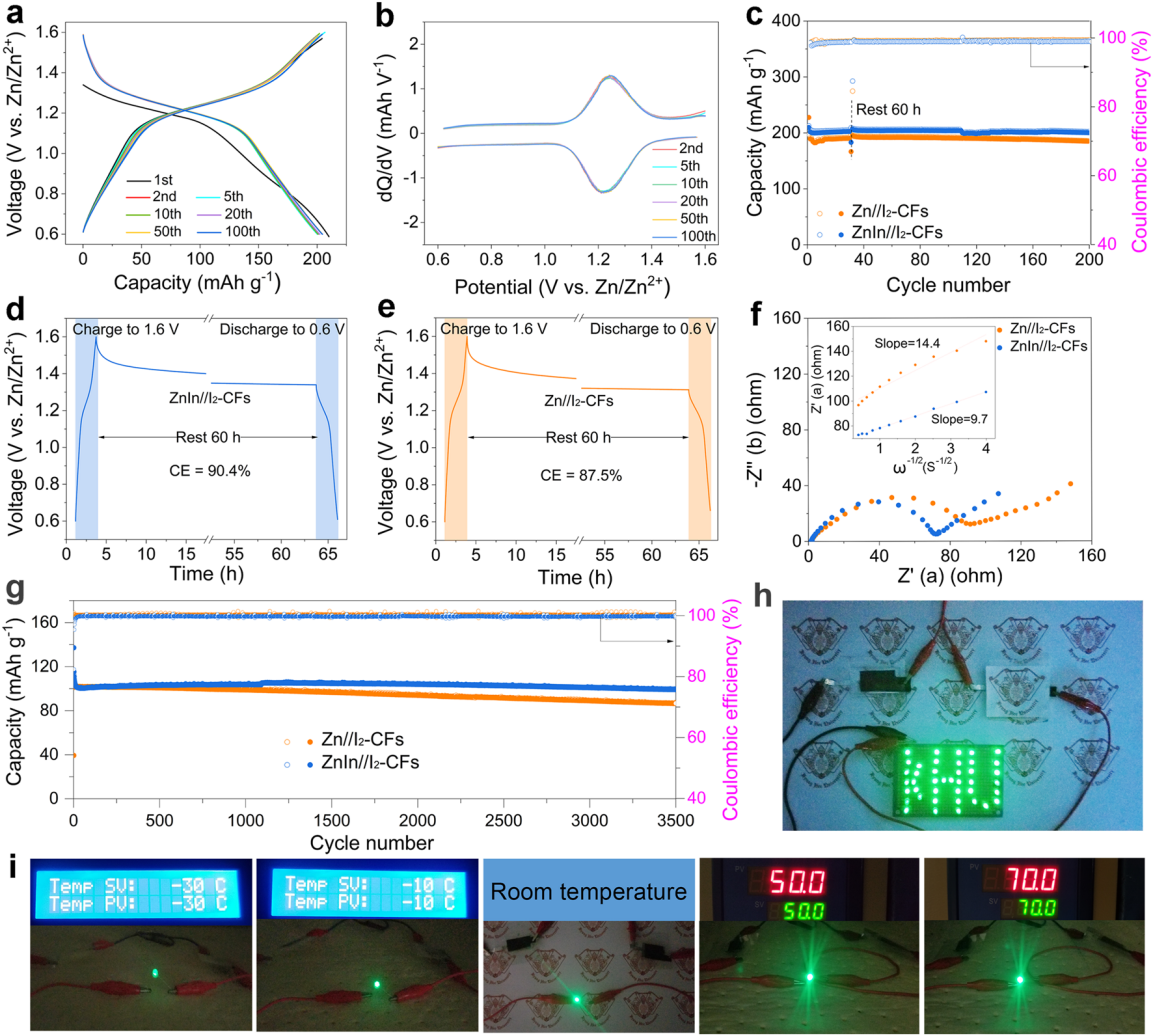
Electrochemical properties of aqueous Zn/I_2_ cells. **a** Charge/discharge profiles of the ZnIn/I_2_-CFs full cell and **b** their corresponding dQ/dV curves. **c** Cycle performance at 0.5 C. Self-discharge curves of the **d** ZnIn/I_2_-CFs and **e** Zn/I_2_-CFs full cells. **f** Nyquist plots, **g** cycle performance at 5.0 C of the full cells, and **h, i** optical photographic images of the quasi-solid-state ZnIn/I_2_-CFs pouch cells powering a LED device

The practical application feasibility of the ZnIn anode was studied by assembling Zn-I_2_ cells. Figure S31a-c and Video S4 show the excellent flexibility of the as-prepared CFs cathode host. The SEM and TEM images (Fig. S31d-f) reveal that the CF has a diameter of approximately 600 nm and exhibit a porous architecture. The porous architecture of the CFs facilitates the anchoring of I_2_ on them. Figure S32a displays that the thickness of the as-prepared CFs is about 200 μm, and the loose structure is conducive to the I_2_ loading and electrolyte infiltration. In addition, the TEM image of the I_2_-CFs architecture and corresponding elemental mappings are further shown in Fig. S32b, indicating the uniform space distribution of C, N, and I within the I_2_-CFs electrode. As shown in Figs. 6a and S33a, the ZnIn/I_2_-CFs and Zn/I_2_-CFs cells deliver the initial discharge capacities of 212.7 and 227.6 mAh g^−1^ at the current density of 0.5 C, and the corresponding CE values are 98.6 and 86.5, respectively. Meanwhile, the subsequent charge/discharge curves of the ZnIn/I_2_-CFs cell display a high overlap, while the Zn/I_2_-CFs battery exhibits slight fluctuations in subsequent cycles, implying excellent stability of the ZnIn/I_2_-CFs cell. The higher initial CE and stable capacity output of the ZnIn/I_2_-CFs cell suggests that the ZnIn anode can more effectively suppress side reactions than the pristine Zn anode. The corresponding d*Q*/d*V* curves are shown in Figs. 6b and S33b. The ZnIn/I_2_-CFs cell shows closer cathodic and anodic peaks than the Zn/I_2_-CFs cell, which indicates that the Zn-In interface provides better electrochemical reaction kinetics and boosts iodine utilization [75, 76]. Besides, the ZnIn/I_2_-CFs cell displays a stable cycle performance compared to the Zn//I_2_-CFs cell at 0.5 C (Fig. 6c). The ZnIn/I_2_ cell shows a better overlap than the Zn/I_2_ cell, indicating a more stable electrochemical performance of the ZnIn anode. The capacity of the Zn/I_2_-CFs cell tends to fade gradually after performing 80 cycles, which is attributed to the surface corrosion and side reactions of the pristine Zn anode. The self-discharge behaviors of the cells were evaluated by operating them for 30 cycles and discharging after 60 h of rest. The ZnIn/I_2_-CFs cell maintains 90.4% of its initial capacity (Fig. 6d), while the Zn/I_2_-CFs cell is 87.5% (Fig. 6e), demonstrating the anticorrosion of the ZnIn anode [13]. In addition, the EIS plots show that the ZnIn/I_2_-CFs cell has lower SEI resistance and charge transfer resistance than the Zn/I_2_-CFs cell in the high-medium frequency region. Meanwhile, the ZnIn/I_2_-CFs cell displays a smaller slope of Z′ versus *ω*^−1/2^ in the low-frequency region, which indicates faster Zn-ion transport kinetics in the ZnIn/I_2_-CFs cell (Fig. 6f). Additionally, the ZnIn/I_2_-CFs cell also shows excellent long-term stable cycling performance at a current density of 5.0 C (Fig. 6g). The capacity of the ZnIn/I_2_-CFs cell is maintained at 99.5 mAh g^−1^ after 3,500 cycles, which was much better than the 86.5 mAh g^−1^ of the Zn/I_2_-CFs cell, indicating the excellent reversibility of the ZnIn/I_2_-CFs cell. The charge–discharge curves of the ZnIn/I_2_ cell show a better overlap than those of the Zn/I_2_ cell, indicating a more stable electrochemical performance of the ZnIn anode (Fig. S34). Additionally, quasi-solid-state devices using gel electrolyte exhibit high mechanical integrity, good reliability, and a wide working temperature range, which have been widely studied in portable and flexible electronic products [77, 78]. To verify the reliability of the prepared ZnIn/I_2_-CFs battery in practical applications, two quasi-solid-state ZnIn/I_2_-CFs pouch cells connected in series can easily power 40 LEDs (Fig. 6h). Meanwhile, it can also provide stable energy output under repeated bending (Fig. S35) and a wide temperature range from − 30 to 70 °C (Figs. 6i and S36), offering new insights for the development of flexible electronic devices.

## Conclusions

In summary, we presented a high-rate and long-life Zn metal anode using Zn metal surface patterning and an ingenious interface design to modulate the Zn deposition behavior. Benefiting from the electron aggregation and zincophilic Zn-In interface in the microchannel, it not only improves the tolerance of the electrode at high current density, but also induces preferential heteroepitaxial Zn deposition in the microchannel and subsequent homoepitaxial Zn deposition on the array surface. Consequently, a dendrite-free Zn metal anode with long-term stability of Zn plating/stripping at high current densities is obtained. Furthermore, the rational design of the ZnIn electrode significantly reduces the nucleation energy barrier of Zn and facilitates the preferential deposition of Zn along the (002) crystallographic plane, thereby improving the compactness of the deposited metal and the overall corrosion resistance of the electrode. In addition, the Zn/I_2_ full cell assembled with the ZnIn anode and I_2_-CFs cathode also delivers higher reversibility and cycle stability than that assembled with a pristine Zn anode. Finally, the stable operation of the quasi-solid-state ZnIn/I_2_-CFs pouch cell and its flexibility tests offer valuable insights for the development of flexible and wearable ZMBs. Therefore, all these results confirm that the surface-patterned metallic Zn anode with the ingenious Zn-In interface design investigated in this study presents a promising strategy for the preparation of high-performance aqueous ZMBs.

## Supplementary Information

Below is the link to the electronic supplementary material.

Supplementary file1 (DOCX 18240 KB)

Supplementary file2 (MP4 34882 KB)

Supplementary file3 (MP4 43378 KB)

Supplementary file4 (MP4 47013 KB)

Supplementary file5 (MP4 2394 KB)
