# Correction: Surface Patterning of Metal Zinc Electrode with an In-Region Zincophilic Interface for High-Rate and Long-Cycle-Life Zinc Metal Anode

**DOI:** 10.1007/s40820-024-01377-6

**Published:** 2024-02-28

**Authors:** Tian Wang, Qiao Xi, Kai Yao, Yuhang Liu, Hao Fu, Venkata Siva Kavarthapu, Jun Kyu Lee, Shaocong Tang, Dina Fattakhova-Rohlfing, Wei Ai, Jae Su Yu

**Affiliations:** 1 Department of Electronics and Information Convergence Engineering, Institute for Wearable Convergence Electronics, Kyung Hee University, Yongin-si, Gyeonggi-do 17104 Republic of Korea; 2 Frontiers Science Center for Flexible Electronics (FSCFE) and Shaanxi Institute of Flexible Electronics (SIFE), Northwestern Polytechnical University (NPU), 127 West Youyi Road, Xi’an, 710072 People’s Republic of China; 3 Institute of Energy and Climate Research: Materials Synthesis and Processing (IEK-1), Forschungszentrum Jülich GmbH, 52425 Jülich, Germany; 4 School of Chemical Engineering, Sungkyunkwan University, 2066 Seobu-Ro, Jangan-Gu, Suwon-si, Gyeonggi-do Republic of Korea

**Correction to: Nano-Micro Letters (2024) 16:112** 10.1007/s40820-024-01327-2

In the supplementary information the following corrections have been carried out:

1. Institute of Energy and Climate Research, Materials Synthesis and Processing,

Forschungszentrum Jülich GmbH, 52425 Jülich, Germany.

Corrected: Institute of Energy and Climate Research: Materials Synthesis and Processing (IEK-1), Forschungszentrum Jülich GmbH, 52425 Jülich, Germany.

2. Fig. S3 Optical photographic image of the P-Zn electrode before and after In powder modification

Corrected: Fig. S3 Optical photographic images of the P-Zn electrode a before and b after In powder modification.

3. Fig. S6 a SEM image and b optical microscopic image of the Zn deposits on the ZnIn electrode with the areal capacities of 15.0 mAh cm-2

Corrected: Fig. S6 a SEM image and b optical microscopic image of the Zn deposits on the ZnIn electrode with the areal capacity of 15.0 mAh cm-2.

4. Fig. S7 SEM images of Zn deposits on the pristine Zn electrode with the current density of 1.0 mA cm-2 for 5 h

Corrected: Fig. S7 a, b SEM images of Zn deposits on the pristine Zn electrode with the current density of 1.0 mA cm-2 for 5 h.

5. Fig. S9 Contact angles of the a pristine Zn, b P-Zn and c P-Zn electrode with the capacities of 3.0, and d pristine ZnIn electrodes and the ZnIn electrode with the capacities of e 3.0 and f 5.0 mAh cm-2.

Corrected: Fig. S9 Contact angles of the a pristine Zn, b P-Zn and c P-Zn electrode with the capacities of 3.0 mAh cm-2, and d pristine ZnIn electrodes and the ZnIn electrode with the capacities of e 3.0 and f 5.0 mAh cm-2.

6. Fig. S17 The EIS curves of the a pristine Zn and b ZnIn electrodes at different temperatures. c The corresponding desolvation activation energy values of the differnet electrodes

Corrected: Fig. S17 EIS curves of the a pristine Zn and b ZnIn electrodes at different temperatures. c Corresponding desolvation activation energy values of the differnet electrodes.

The original article has been corrected.

