# Supplementary material for: Surface Patterning of Metal Zinc Electrode with an In-Region Zincophilic Interface for High-Rate and Long-Cycle-Life Zinc Metal Anode
Source: Nanomicro Lett. 2024 Feb 9;16:112. doi: 10.1007/s40820-024-01327-2 (PMC10858015; doi:10.1007/s40820-024-01327-2)
Supplement: Supplementary file 1 — Supplementary file1 (DOCX 18240 KB) [file 40820_2024_1327_MOESM1_ESM.docx]

Supporting Information for

**Surface Patterning of Metal Zinc Electrode with an In-Region Zincophilic Interface for High-Rate and Long-Cycle-Life Zinc Metal Anode**

Tian Wang^1^, Qiao Xi^2^, Kai Yao^3^, Yuhang Liu^2^, Hao Fu^4^, Venkata Siva Kavarthapu^1^, Jun Kyu Lee^1^, Shaocong Tang^1^, Dina Fattakhova-Rohlfing^3^, Wei Ai^2,^ *, Jae Su Yu^1,^ *

^1^ Department of Electronics and Information Convergence Engineering, Institute for Wearable Convergence Electronics, Kyung Hee University, Yongin-si, Gyeonggi-do 17104, Republic of Korea

^2^ Frontiers Science Center for Flexible Electronics (FSCFE) and Shaanxi Institute of Flexible Electronics (SIFE), Northwestern Polytechnical University (NPU), 127 West Youyi Road, Xi'an 710072, People’s Republic of China

^3^ Institute of Energy and Climate Research: Materials Synthesis and Processing (IEK-1), Forschungszentrum Jülich GmbH, 52425 Jülich, Germany

^4^ School of Chemical Engineering, Sungkyunkwan University, 2066 Seobu-ro, Jangan-gu, Suwon-si, Gyeonggi-do, Republic of Korea

*Corresponding author. E-mail: iamwai@nwpu.edu.cn (W. Ai), jsyu@khu.ac.kr (J. S. Yu)

**Supplementary Figures and Table**


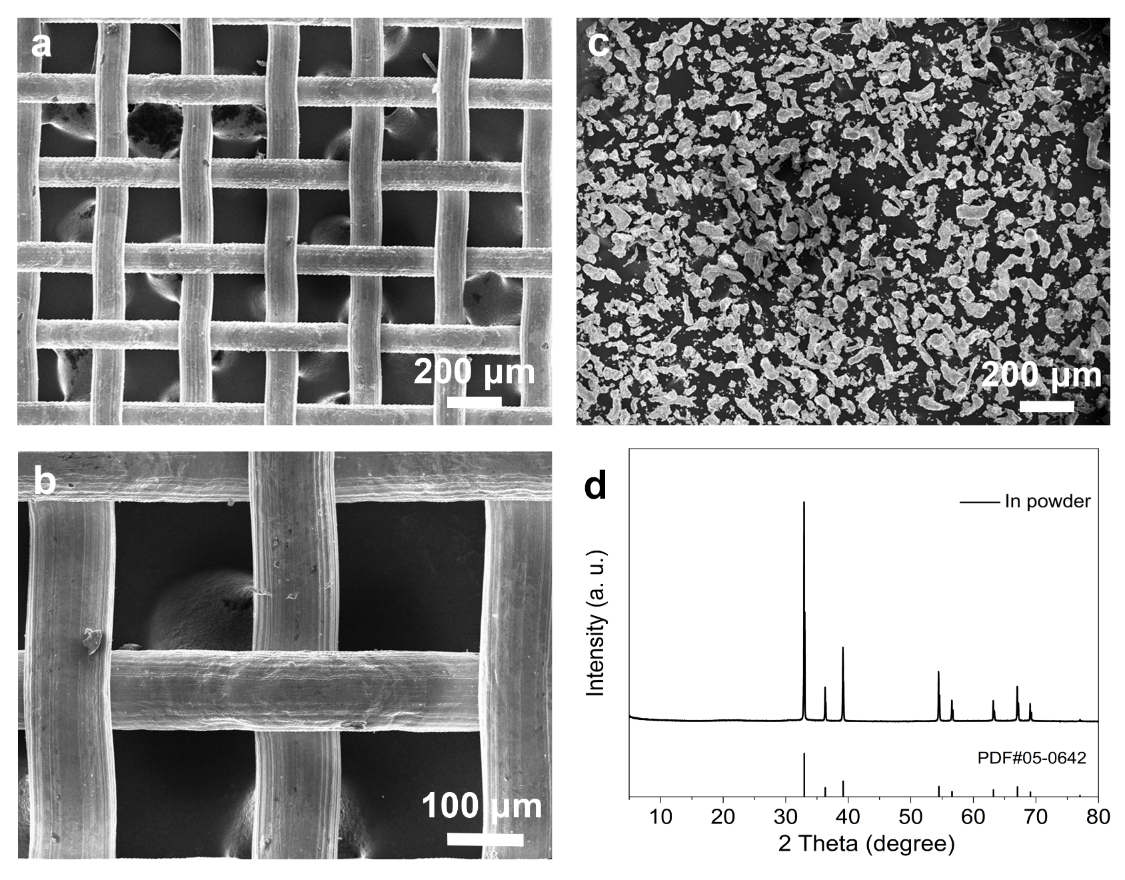


**Fig. S1** SEM images of the **a, b** stainless steel meshes and **c** In powder. **d** XRD patterns of the In powder


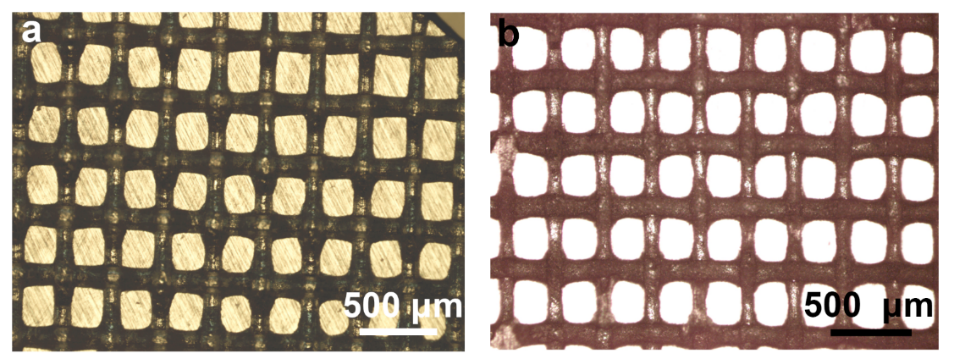


**Fig. S2** Optical microscopic images of **a** the Zn electrodes after the rolling process and **b** the pristine ZnIn electrode


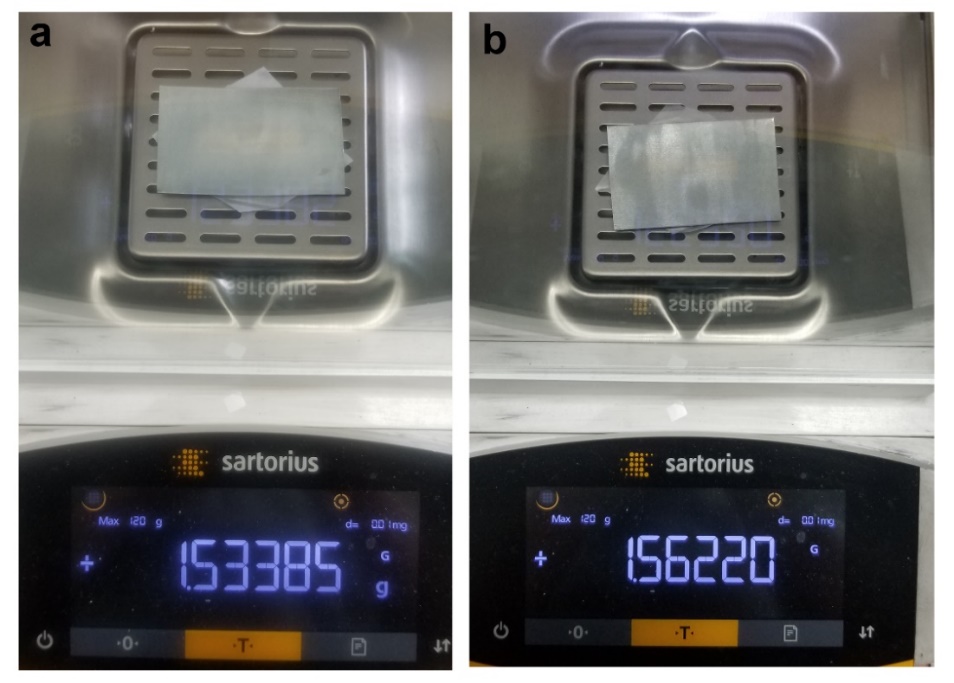


**Fig. S3** Optical photographic images of the P-Zn electrode **a** before and **b** after In powder modification


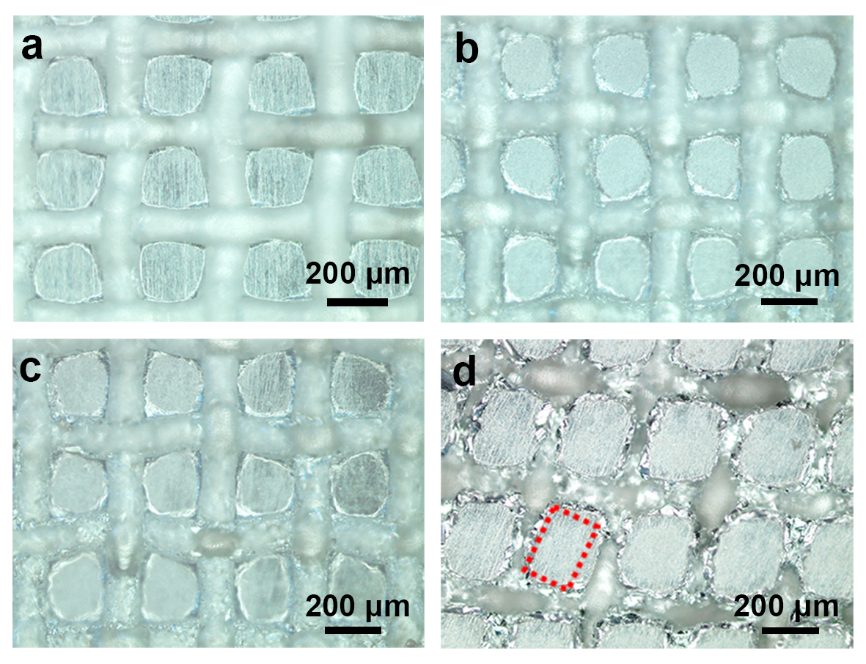


**Fig. S4** Optical microscopic images of the Zn deposits on the ZnIn electrode with the areal capacities of **a** 0 mAh cm^-2^, **b** 3.0 mAh cm^-2^, **c** 5.0 mAh cm^-2^, and **d** 10.0 mAh cm^-2^


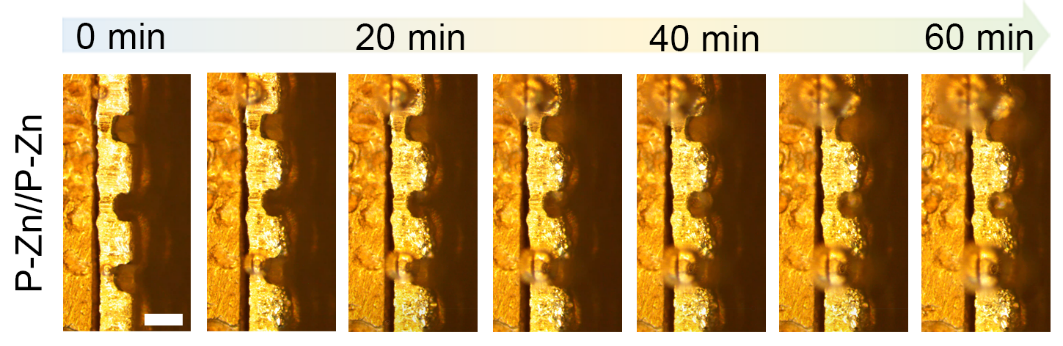


**Fig. S5** In situ optical microscopic images of the Zn deposition on the P-Zn electrode. Scale bar: 100 μm


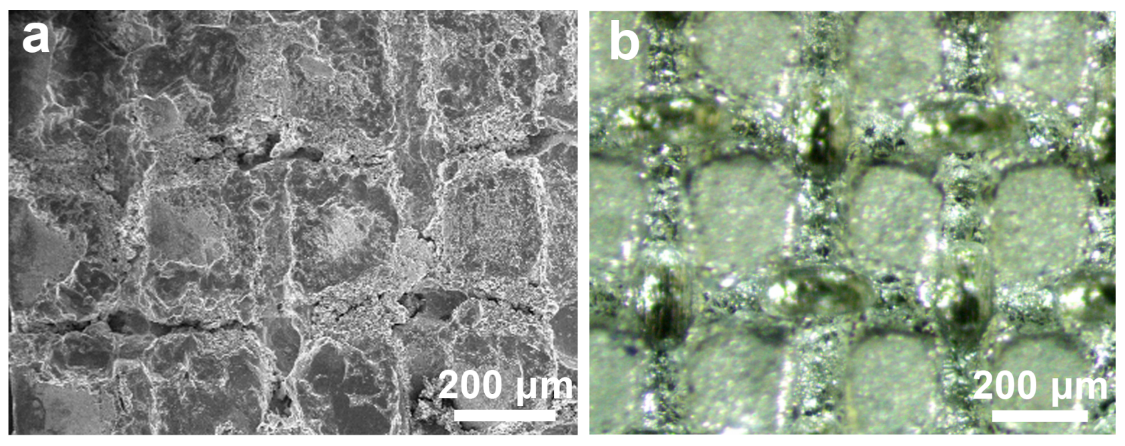


**Fig. S6** **a** SEM image and **b** optical microscopic image of the Zn deposits on the ZnIn electrode with the areal capacity of 15.0 mAh cm^-2^


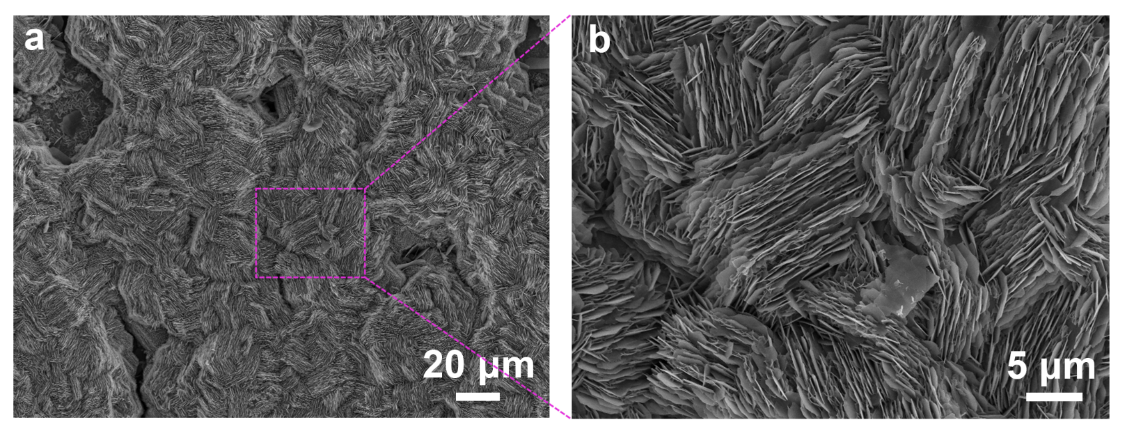


**Fig. S7 a, b** SEM images of Zn deposits on the pristine Zn electrode with the current density of 1.0 mA cm^-2^ for 5 h


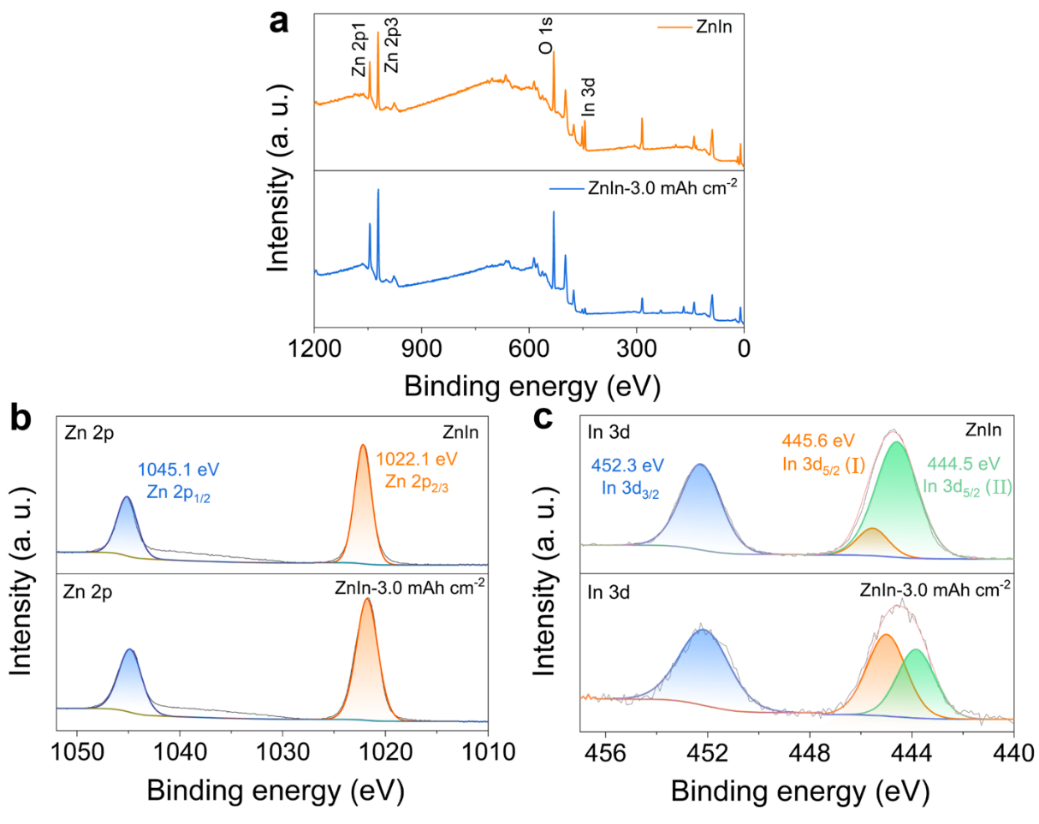


**Fig. S8** **a** XPS full survey scan spectra and high-resolution core-level spectra of **b** Zn 2p and **c** In 3d for the pristine ZnIn electrode and the ZnIn electrode after Zn metal deposition


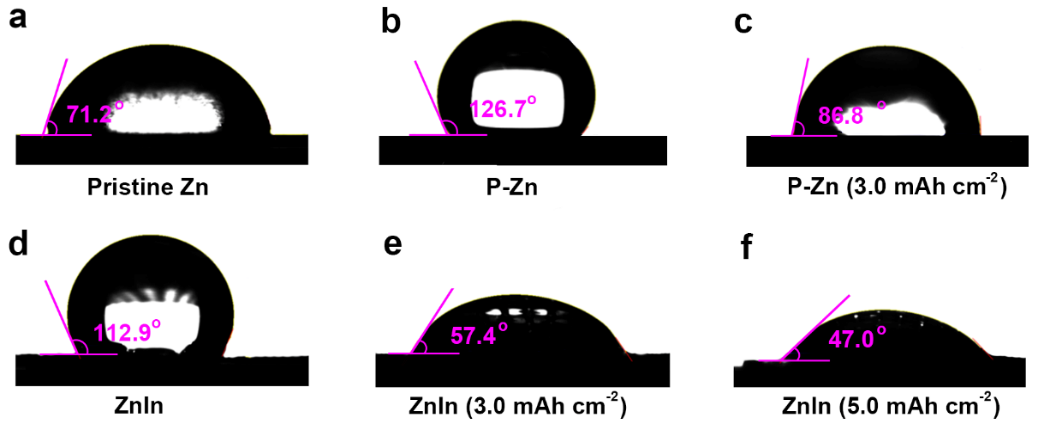


**Fig. S9** Contact angles of the **a** pristine Zn, **b** P-Zn and **c** P-Zn electrode with the capacities of 3.0 mAh cm^-2^, and **d** pristine ZnIn electrodes and the ZnIn electrode with the capacities of **e** 3.0 and **f** 5.0 mAh cm^-2^


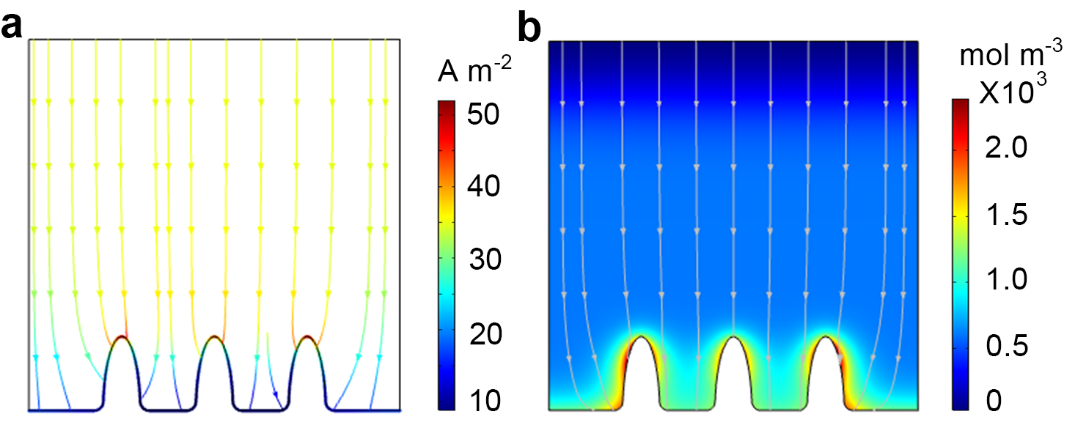


**Fig. S10** Simulation results of **a** the current density and **b** the Zn ion concentration distribution on the pristine Zn electrode surface


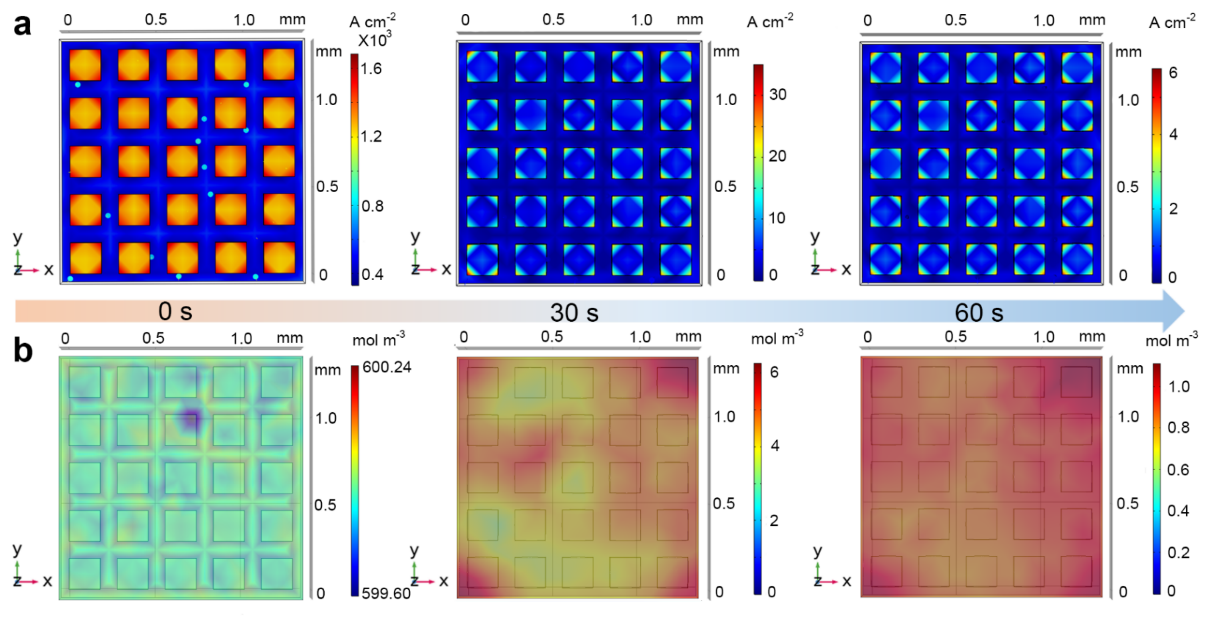


**Fig. S11** 2D view of the evolution process of **a-c** the current density and **d-f** the Zn ion concentration distribution on the ZnIn electrode surface


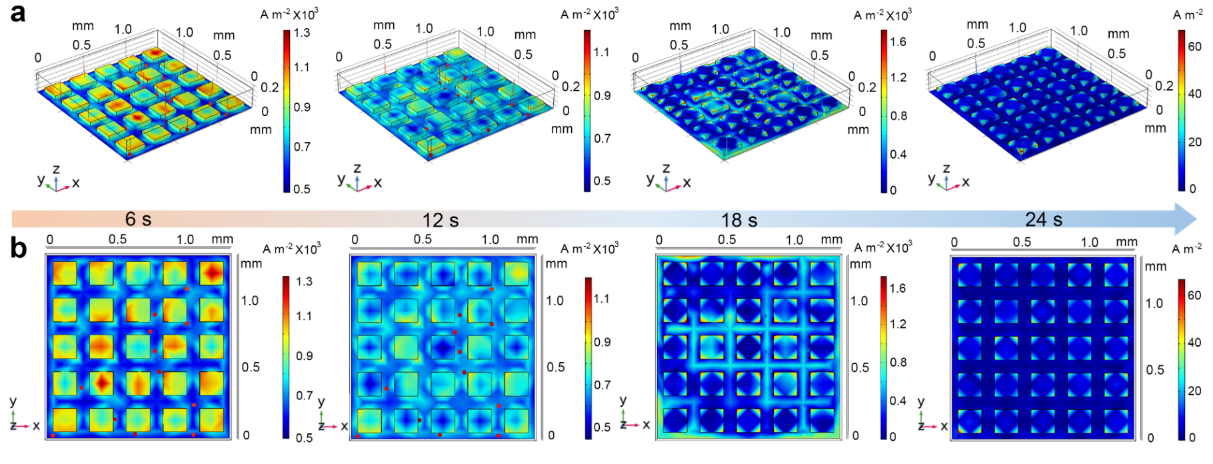


**Fig. S12** **a** 3D view of the evolution process of the current density on the ZnIn electrode surface and **b** its corresponding 2D view

**
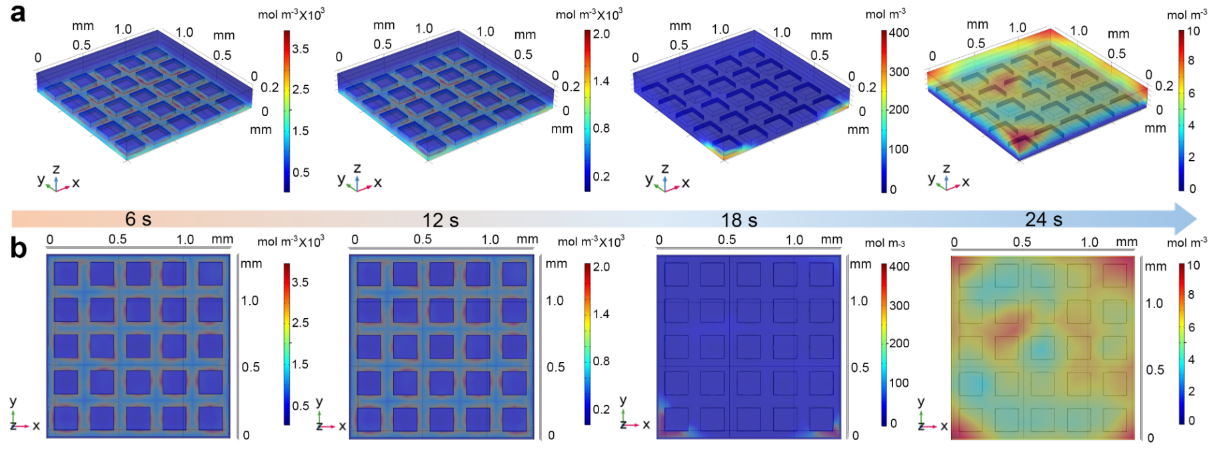
**

**Fig. S13** **a** 3D view of the evolution process of the Zn ion concentration on the ZnIn electrode surface and **b** its corresponding 2D view


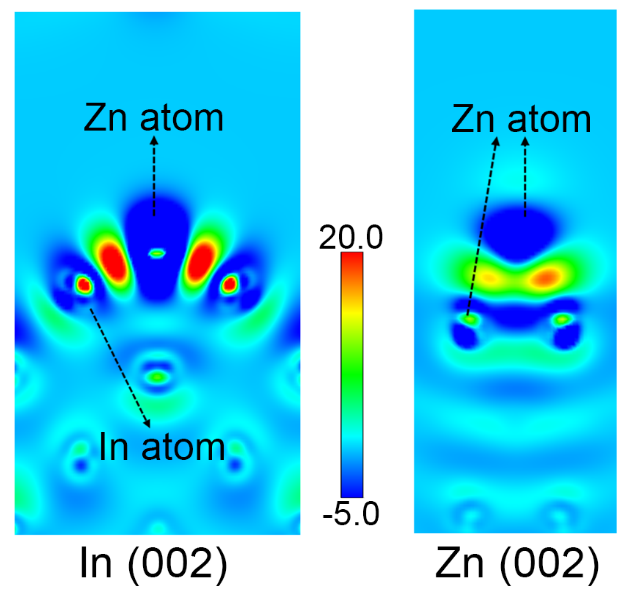


**Fig. S14** Slice of the electron density difference map of the Zn atom on the In (002) and Zn (002) planes, respectively


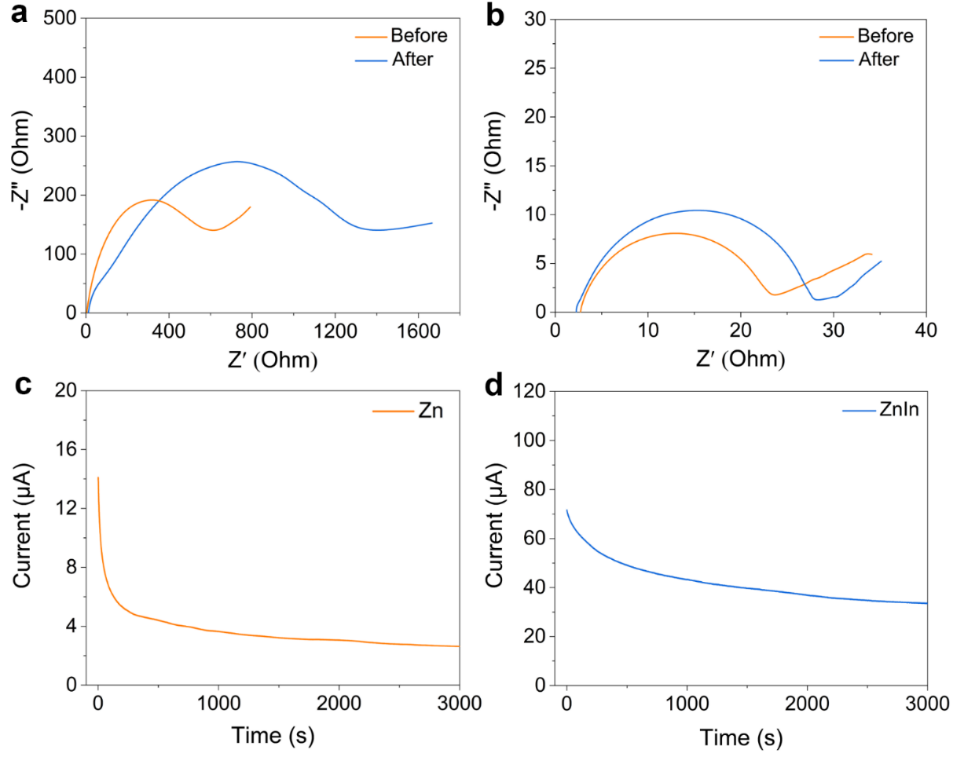


**Fig. S15** Nyquist plots of the **a** Zn and **b** ZnIn symmetric batteries before and after polarization test and the corresponding current-time curves of the **c** Zn and **d** ZnIn symmetric cells under the constant voltage polarization of 5 mV.

The Zn ions transference number was obtained by the Evans’ method [S1, S2]:

$$t_{{Zn}^{2+}}=\frac{I_{S}\left( \boldsymbol{\Delta}V\boldsymbol{-}I_{0}R_{0} \right)}{I_{0}(\Delta V-I_{S}R_{S})}$$

where *I_0_* and *I*_s_ are the currents of the initial and steady state, respectively and *R_s_* and *R_s_* represent the corresponding resistances, respectively. The Δ*V* means the applied voltage polarization.


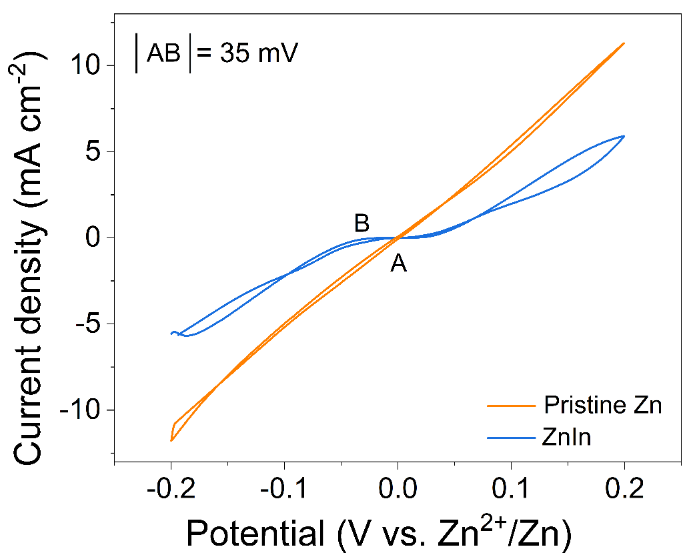


**Fig. S16** CV curves of Zn plating/stripping on pristine Zn and ZnIn electrodes


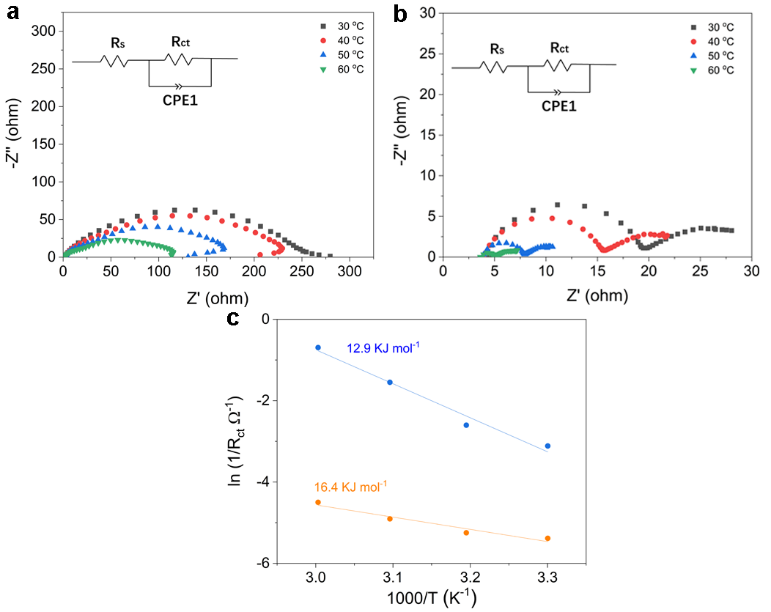


**Fig. S17** EIS curves of the **a** pristine Zn and **b** ZnIn electrodes at different temperatures. **c** Corresponding desolvation activation energy values of the differnet electrodes


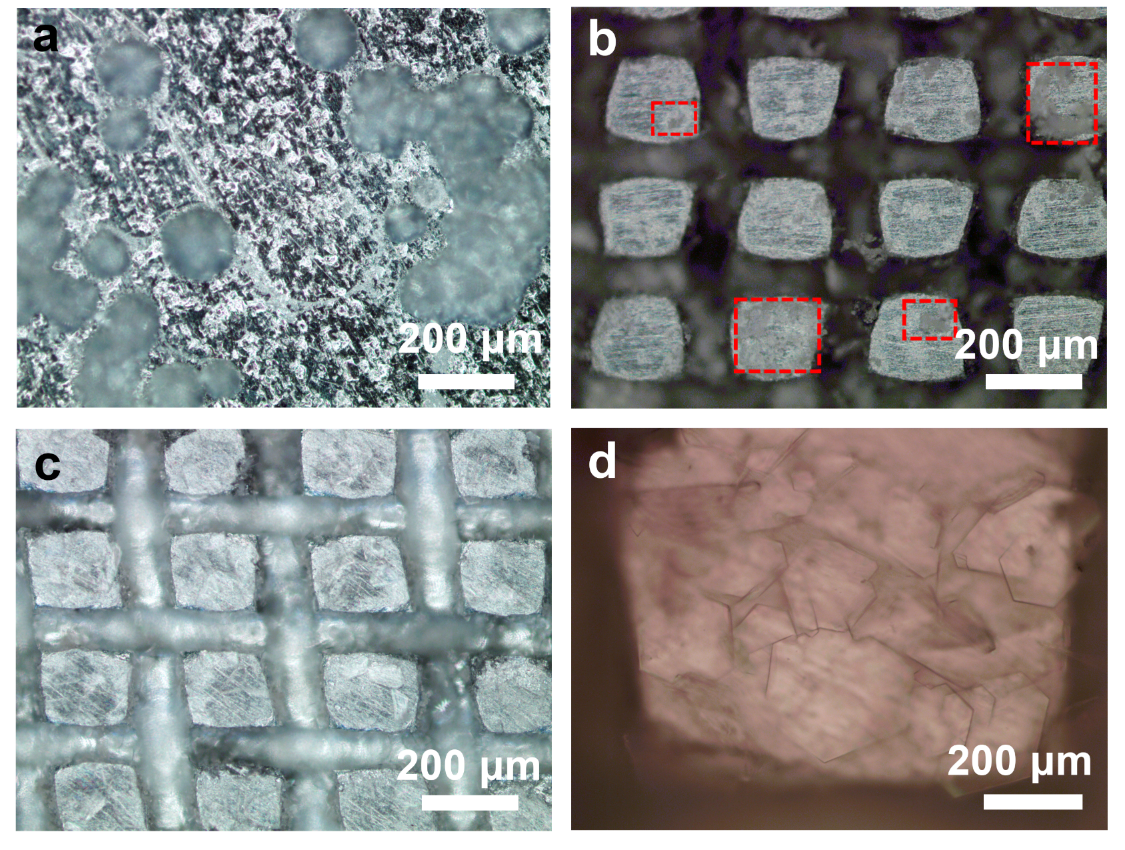


**Fig. S18** Optical microscopic images of the **a** pristine Zn and **b** P-Zn, and **c, d** ZnIn electrodes after cycling 100 h at the current density of 1.0 mA cm^-2^ with the area capacity of 1.0 mA cm^-2^


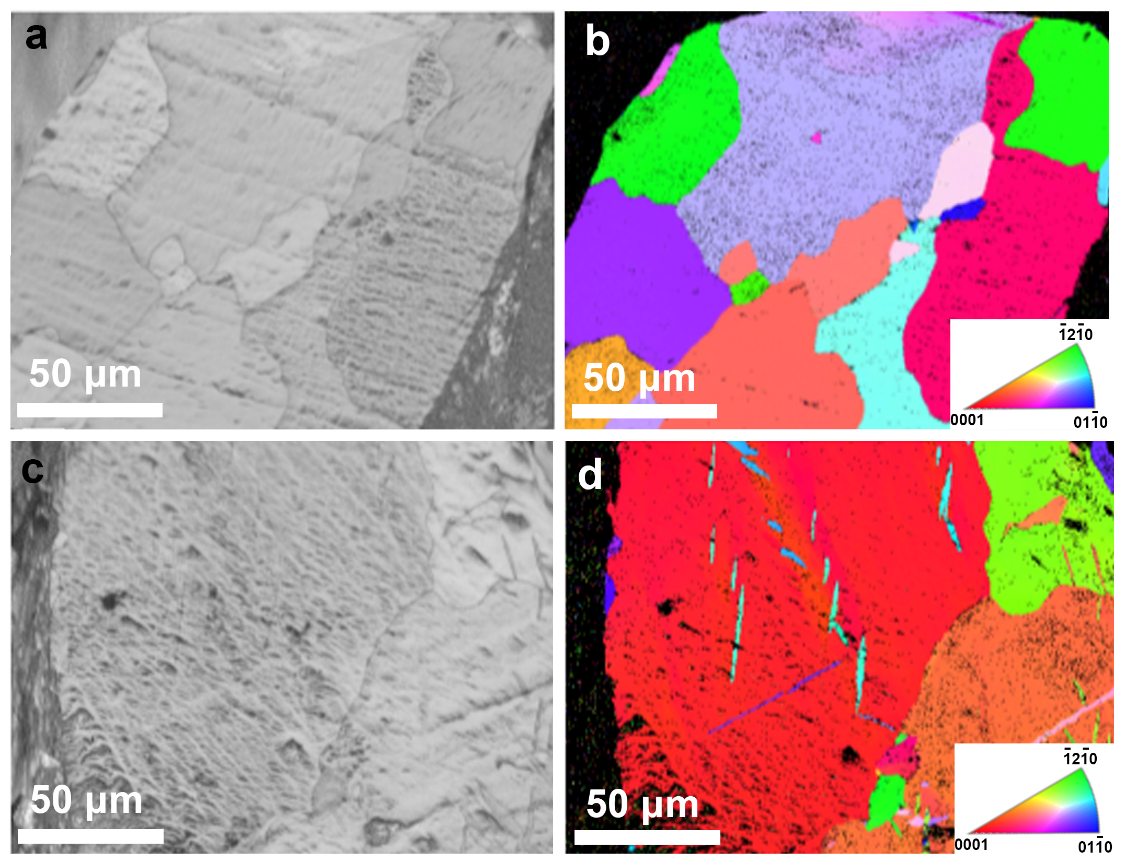


**Fig. S19** Band contrast image and its corresponding EBSD mapping of the ZnIn electrode **a**, **b** before and **c**, **d** after cycling 100 h


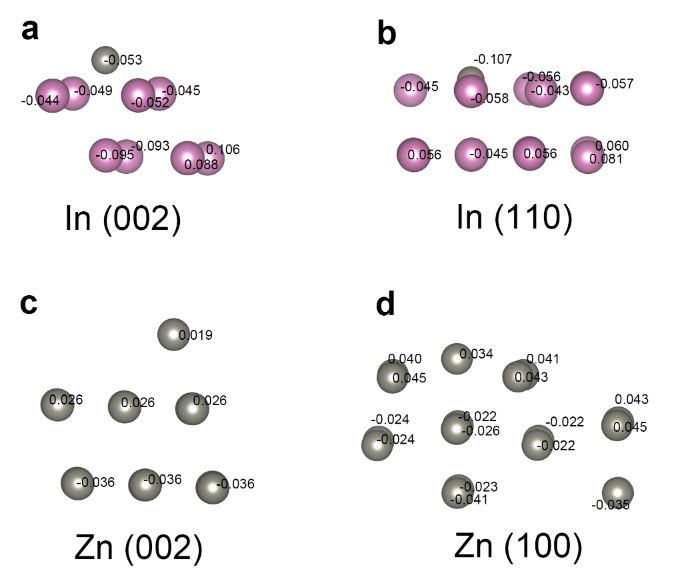


**Fig. S20** Mulliken charge distributions of the Zn atom on the **a** In (002), **b** In (110), **c** Zn (002), and **d** Zn (100) planes, respectively


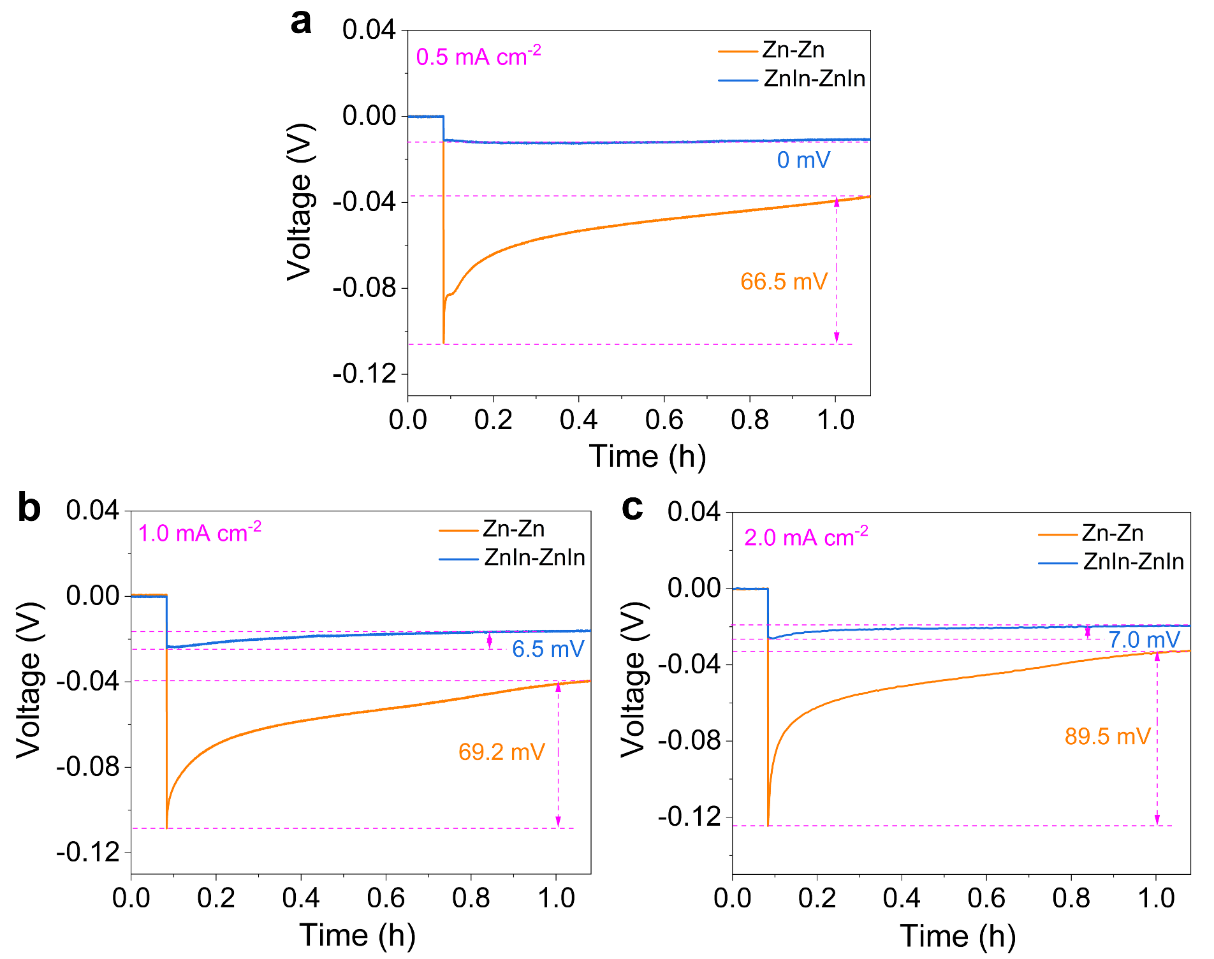


**Fig. S21** Voltage profiles of Zn deposition on the pristine Zn and ZnIn electrodes at **a** 0.5 mA cm^-2^, **b** 1.0 mA cm^-2^, and **c** 2.0 mA cm^-2^


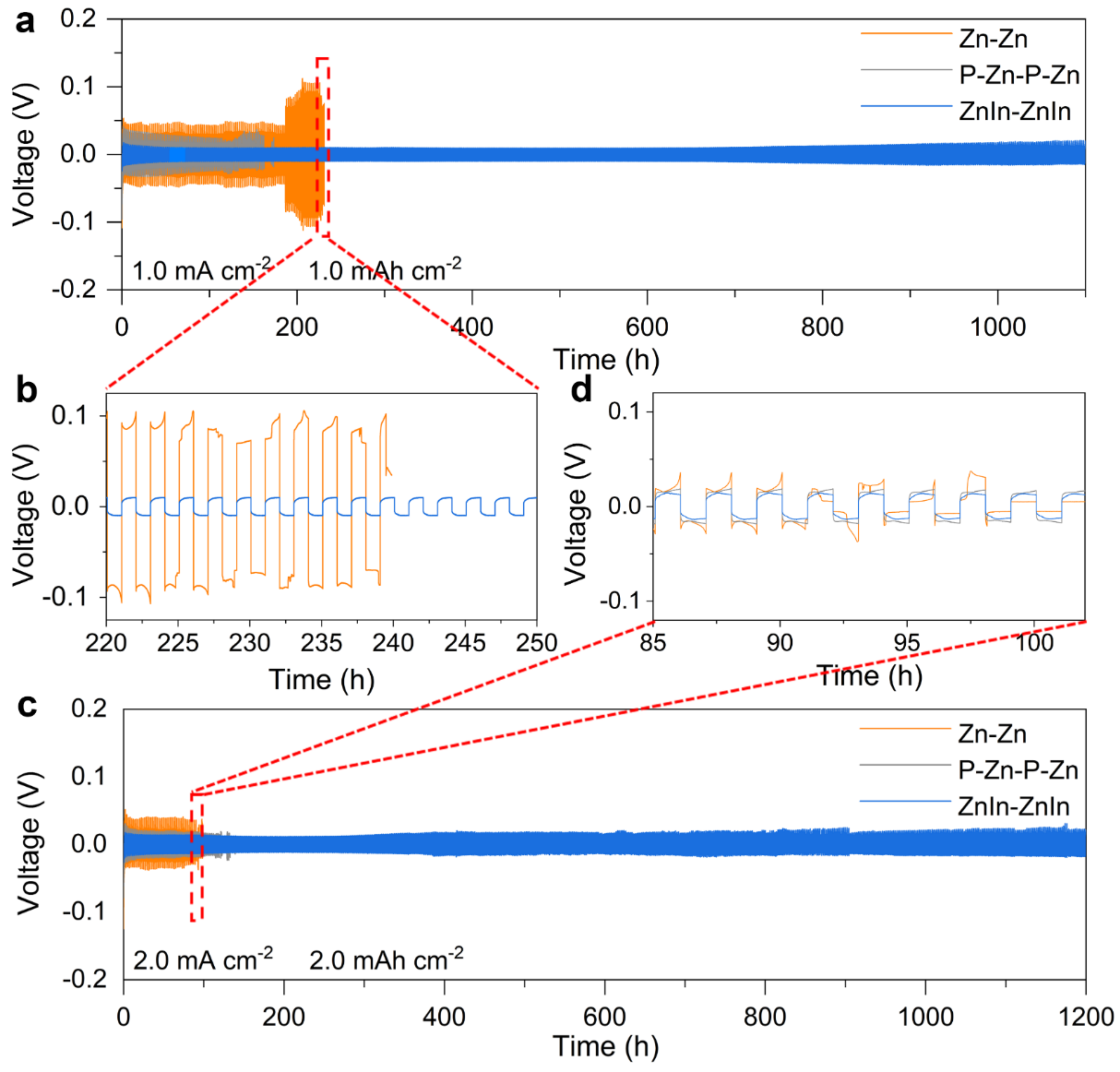


**Fig. S22** Voltage profiles of pristine Zn, P-Zn, and ZnIn symmetric cells with plating/strapping conditions of **a** 1.0 mA cm^-2^ and 1.0 mAh cm^-2^ and **c** 2.0 mA cm^-2^ and 2.0 mAh cm^-2^. **b, d** Their corresponding magnified curves at specific time in **a, c,** respectively

**
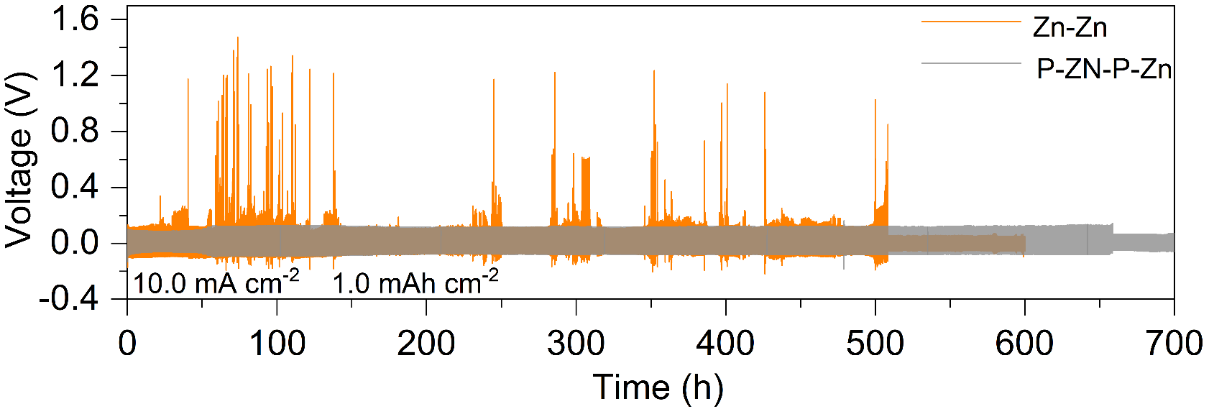
**

**Fig. S23** Voltage profiles of the pristine Zn and P-Zn symmetric cells with plating/strapping conditions of 10.0 mA cm^-2^ and 1.0 mAh cm^-2^


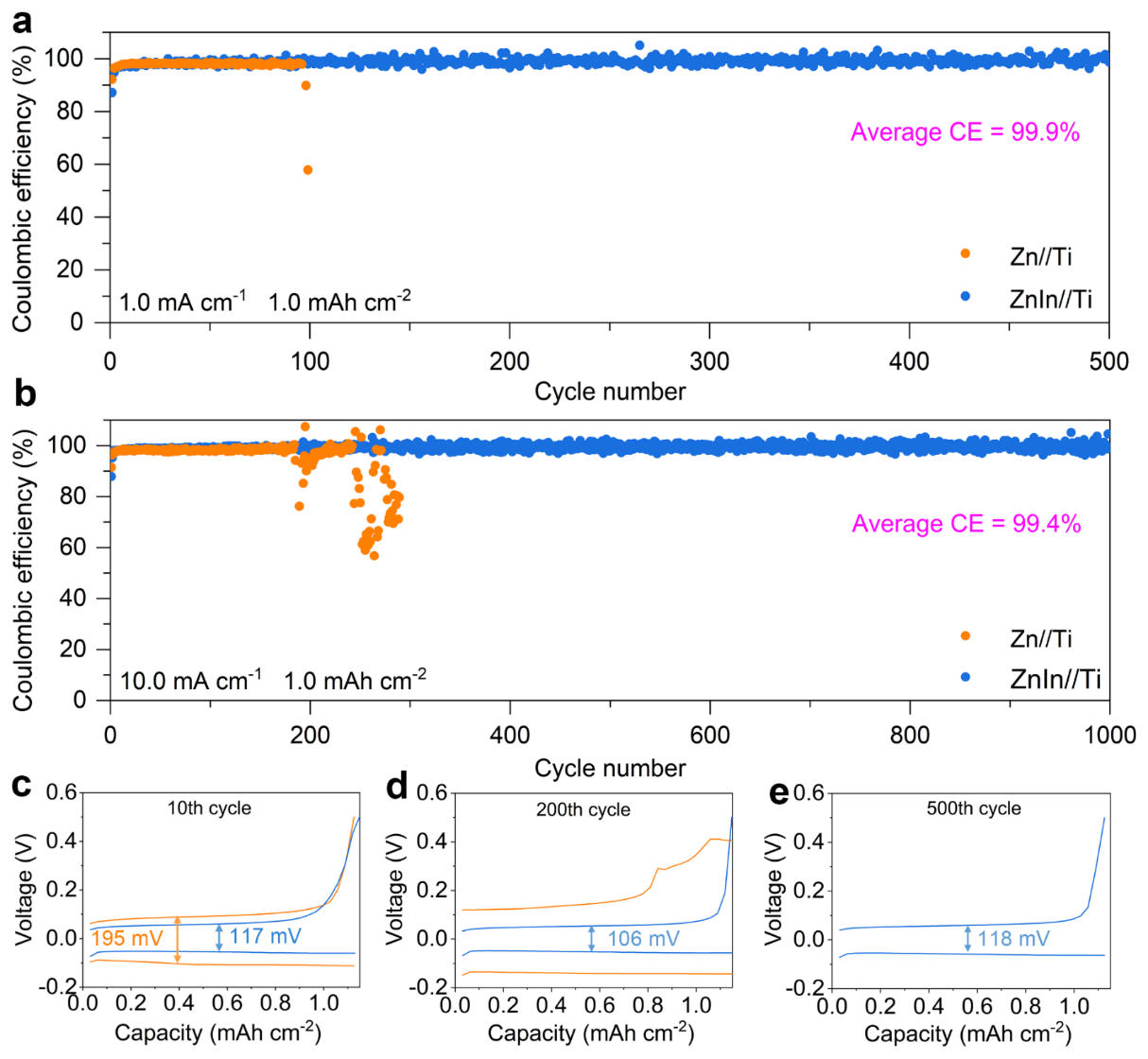


**Fig. S24** Coulombic efficiency of the Zn plating/stripping on Ti foil at **a** 1.0 mA cm^-2^ and 1.0 mAh cm^-2^ and **b** 10.0 mA cm^-2^ and 1.0 mAh cm^-2^. **c-e** Plating and stripping voltage profiles of Zn//Ti cell at 10.0 mA cm^-2^ and 1.0 mAh cm^-2^

**
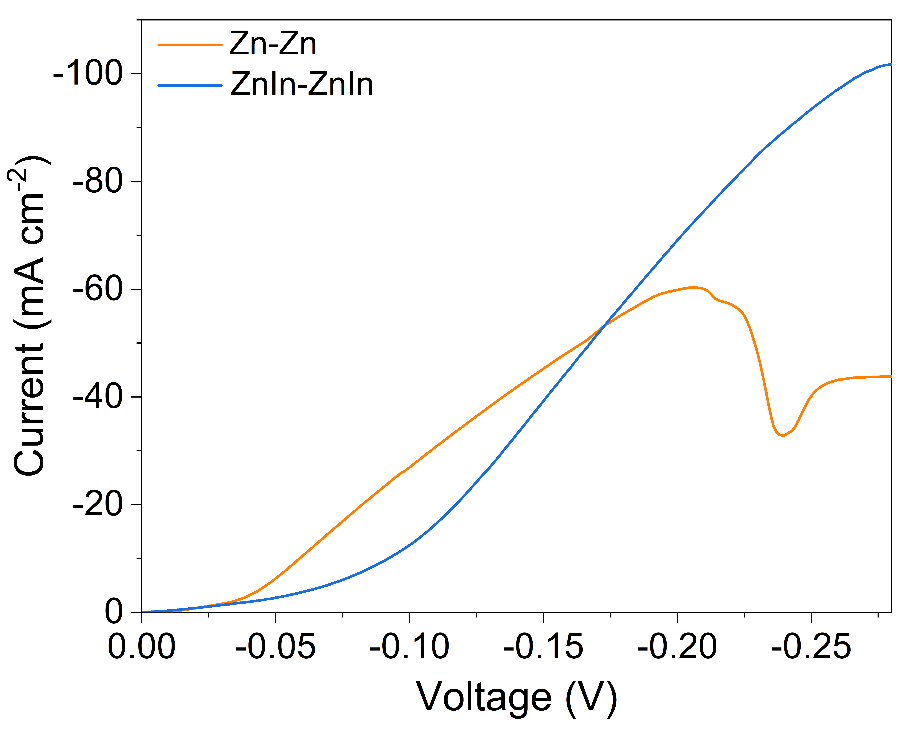
**

**Fig. S25** Voltammetry of the pristine Zn and ZnIn symmetric cells at a scan rate of 1.0 mV s^-1^


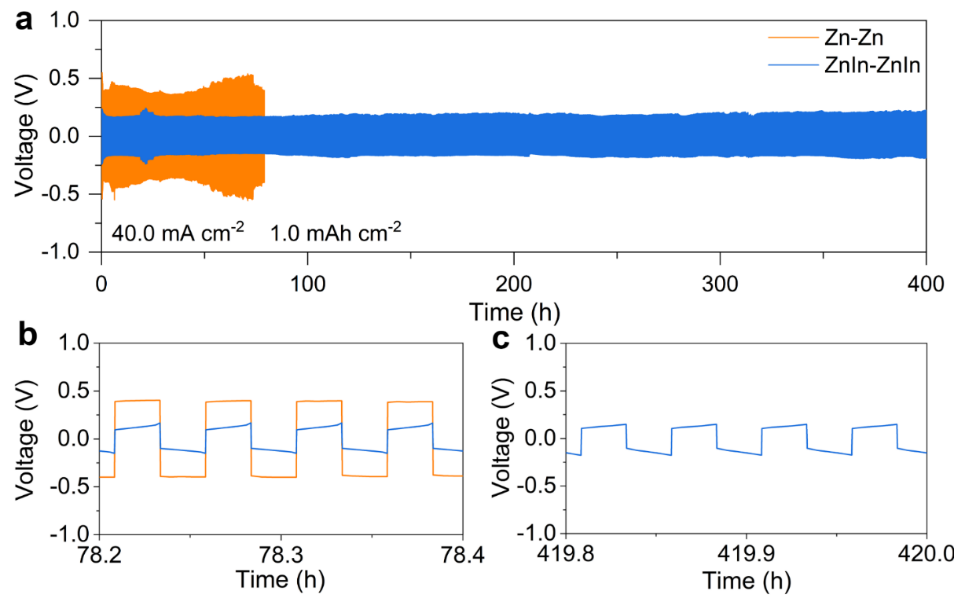


**Fig. S26** **a** Voltage profiles of the pristine Zn and ZnIn symmetric cells with plating/strapping conditions of 40.0 mA cm^-2^ and 1.0 mAh cm^-2^ and **b, c** magnified voltage-time curves at different times


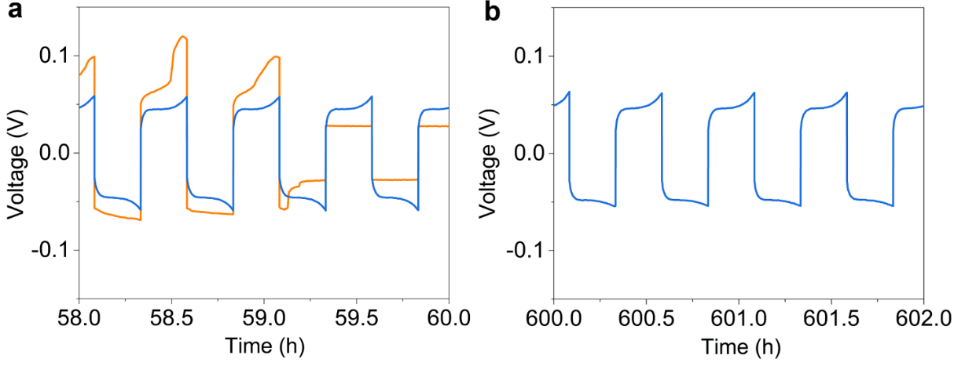


**Fig. S27** Magnified voltage-time curves at different times in Fig. 4c


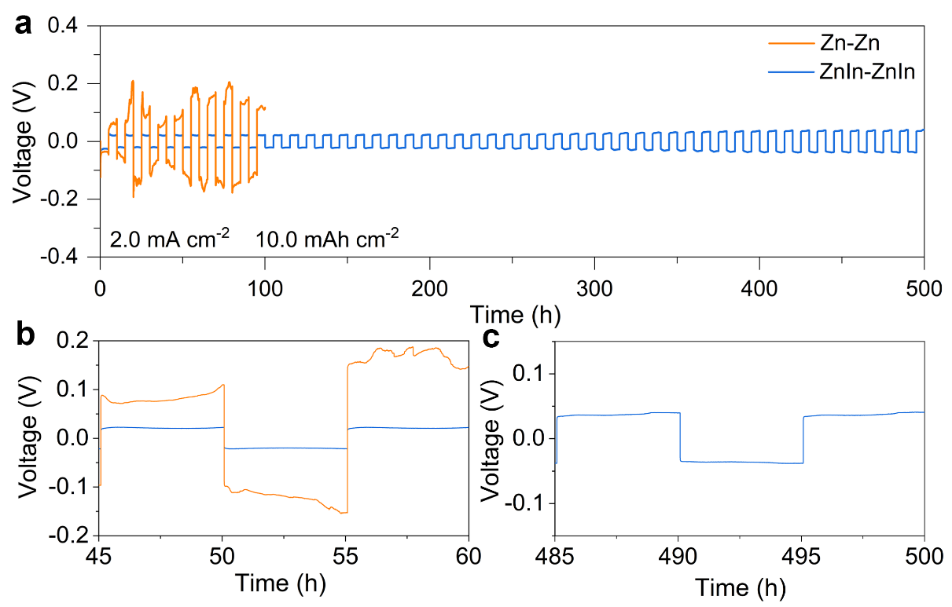


**Fig. S28** **a** Voltage profiles of the pristine Zn and ZnIn symmetric cells with plating/strapping conditions of 2.0 mA cm^-2^ and 10.0 mAh cm^-2^ and **b, c** magnified voltage-time curves at different times


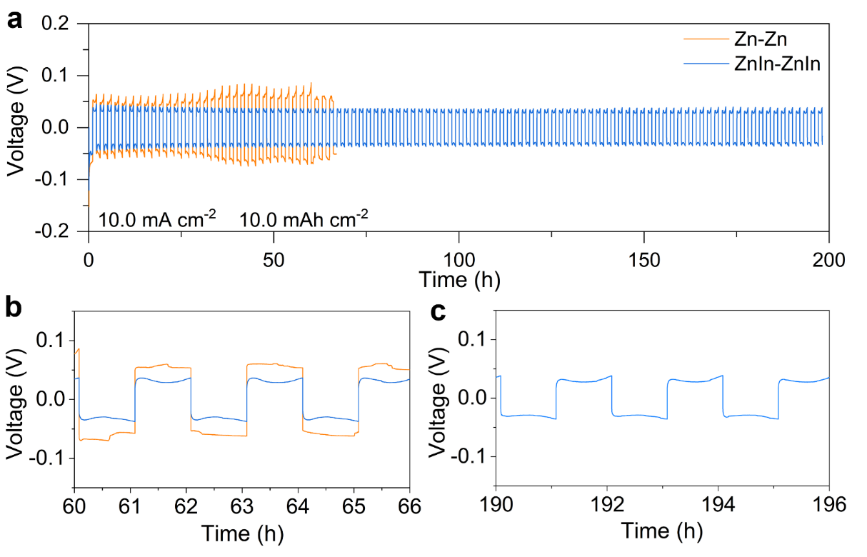


**Fig. S29** **a** Voltage profiles of the pristine Zn and ZnIn symmetric cells with plating/strapping conditions of 10.0 mA cm^-2^ and 10.0 mAh cm^-2^ and **b, c** magnified voltage-time curves at different times


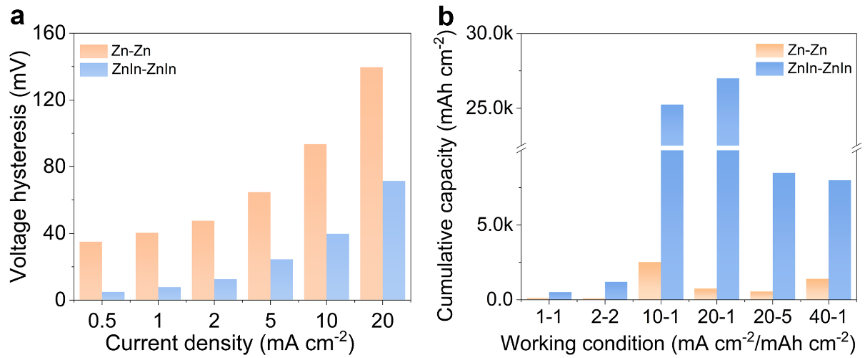


**Fig. S30** Comparison of **a** the voltage polarization from rate performance and **b** the cumulative capacity of the pristine Zn and ZnIn symmetric cells at various working conditions

**Table S****1** Performance comparison of different modification strategies for Zn anode.

| Materials | Method/Mechanism | Current density  (mA cm^-2^) | Areal  capacity  (mAh cm^-2^) | Time (h) | Refs. |
| --- | --- | --- | --- | --- | --- |
| Hydroxyl-rich  silica ion sieve | Separator modification | 1.0 | 1.0 | 3400 | [S3] |
|  |  | 10.0 | 1.0 | 2550 |  |
| TiN | Protective  coating layer | 0.5 | 0.5 | 2800 | [S4] |
|  |  | 1.0 | 1.0 | 2300 |  |
|  |  | 2.0 | 2.0 | 1050 |  |
| 3D porous Ti | Nanoporous host | 1.0 | 1.0 | 2000 | [S5] |
|  |  | 10.0 | 0.5 | 500 |  |
| 3D intertwined  bacterial cellulose | In situ  self-assembly | 0.5 | 0.25 | 3000 | [S6] |
|  |  | 5.0 | 2.5 | 570 |  |
|  |  | 5.0 | 5.0 | 300 |  |
| Zinc phosphate | Hydrothermal reaction | 1.0 | 1.0 | 469 | [S7] |
|  |  | 5.0 | 1.0 | 1976 |  |
|  |  | 10.0 | 1.0 | 500 |  |
| Sulfonate-rich | Ion-exchange layer | 1.0 | 1.0 | 600 | [S8] |
|  |  | 2.0 | 2.0 | 230 |  |
| Cross-linked  gelatin | Artificial interface layer | 1.0 | 1.0 | 4000 | [S9] |
|  |  | 2.0 | 2.0 | 200 |  |
| Betaine | Electrolyte additive | 0.5 | 0.5 | 4200 | [S10] |
|  |  | 2.0 | 2.0 | 830 |  |
| Carbonyl-containing  Layer | Ion redistributor  and functional protective interphase | 1.0 | 0.25 | 5000 | [S11] |
|  |  | 4.0 | 1.0 | 2100 |  |
|  |  | 10.0 | 2.5 | 820 |  |
| ZnO/C  nanoparticles | Host | 10.0 | 1.0 | 400 | [S12] |
|  |  | 20.0 | 1.0 | 150 |  |
| Bi | Termodynamics inertia and kinetics zincophilia | 2.0 | 1.0 | 1700 | [S13] |
|  |  | 5.0 | 2.0 | 1500 |  |
|  |  | 10.0 | 1.0 | 2000 |  |
|  |  | 10.0 | 5.0 | 310 |  |
| C/Cu nanocomposite decoration layer | Functional ultrathin separators | 1.0 | 0.5 | 2000 | [S14] |
|  |  | 5.0 | 2.5 | 650 |  |
|  |  | 10.0 | 2.0 | 600 |  |
| Zn(NO_3_)_2_ 6H_2_O and (NH_4_)_2_HPO_4_ | Conversion  coating | 5.0 | 1.25 | 2000 | [S15] |
|  |  | 20.0 | 5.0 | 470 |  |
| Graphdiyne | Atomic electrode | 10.0 | 1.0 | 3200 | [S16] |
|  |  | 30.0 | 1.0 | 250 |  |
| Metal-organic  complex interphase | In situ complexing of metal-phytic acid | 0.5 | 0.25 | 2000 | [S17] |
|  |  | 5.0 | 2.5 | 1750 |  |
| Lanthanum nitrate | Electrolyte additive | 1.0 | 1.0 | 1200 | [S18] |
|  |  | 10.0 | 5.93 | 160 |  |
| Zn anode with 0.3 mAh cm^-2^ perdeposited layer | Stable zinc metal electrode surface morphologies | 5.0 | 1.0 | 1000 | [S19] |
|  |  | 7.5 | 1.0 | 700 |  |
|  |  | 10.0 | 1.0 | 500 |  |
| Poled ferroelectric coating layer | Deconcentrate and  self-accelerate ion migration | 1.0 | 1.0 | 4000 | [S20] |
|  |  | 10..0 | 2.0 | 1250 |  |
|  |  | 20.0 | 2.0 | 625 |  |
|  |  | 40.0 | 2.0 | 225 |  |
| Hexamethylenetetramine | Electrolyte additive | 5.0 | 1.0 | 4000 | [S21] |
|  |  | 5.0 | 5.0 | 590 |  |
| Yolk-shell  microspheres film | Artificial interface layer | 1.0 | 0.5 | 3800 | [S22] |
|  |  | 10.0 | 1.0 | 4000 |  |
| ZnIn anode | Surface patterning and zincphilic interface design | 1.0 | 1.0 | 1020 | This work |
|  |  | 2.0 | 2.0 | 1200 |  |
|  |  | 10.0 | 1.0 | 5050 |  |
|  |  | 20.0 | 1.0 | 2700 |  |
|  |  | 20.0 | 5.0 | 850 |  |
|  |  | 40.0 | 1.0 | 400 |  |


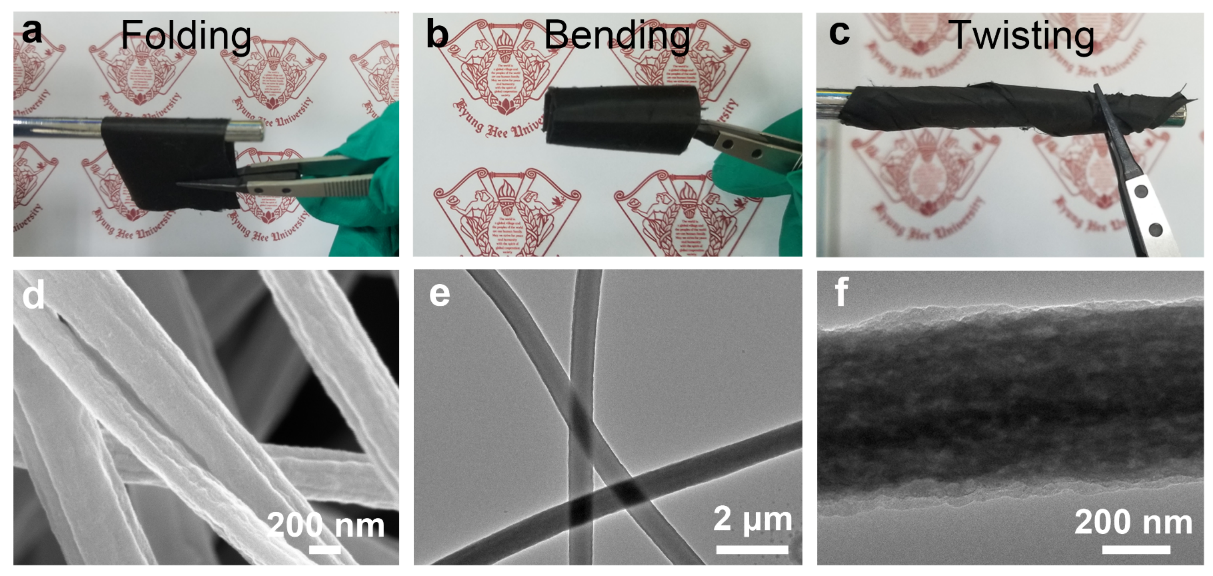


**Fig. S31** Photographic images of the obtained CFs host at **a** folding, **b** bending, and **c** twisting states. **d** SEM and **e, f** TEM images of the CFs


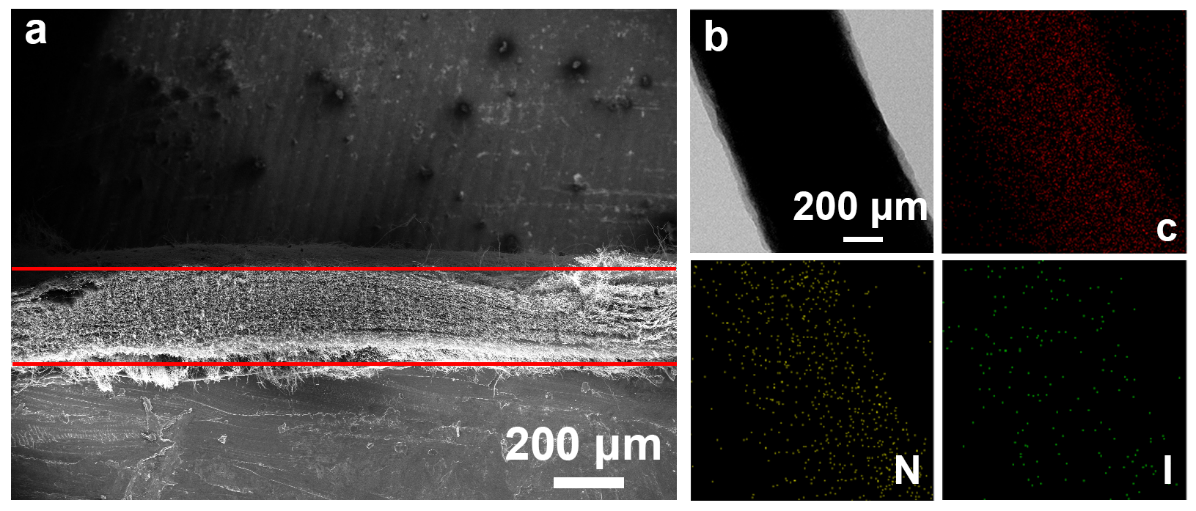


**Fig. S32** **a** Cross-sectional SEM image of the CFs. **b** TEM image and the corresponding EDS mapping images of the I_2_-CFs electrode


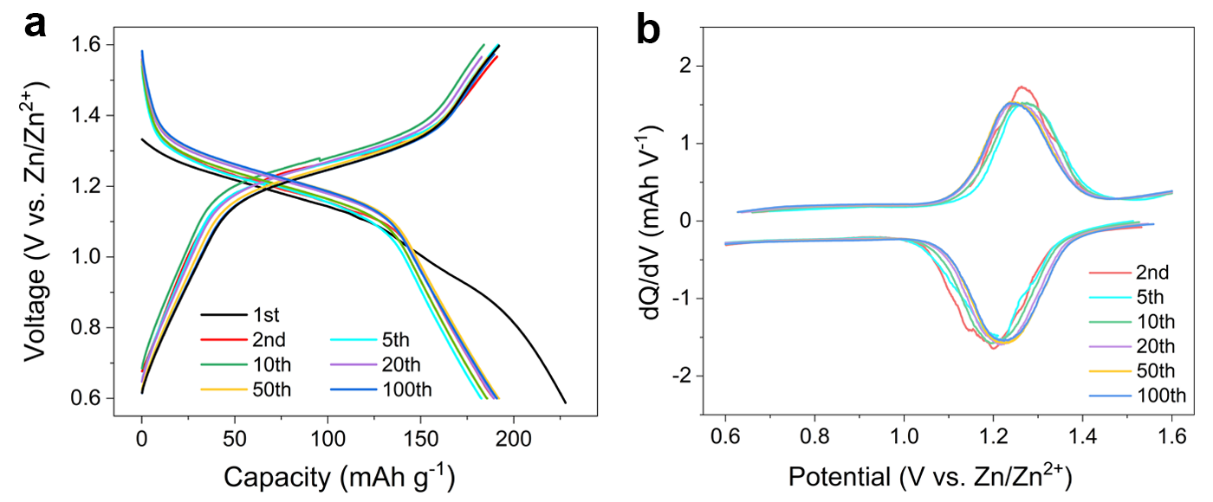


**Fig. S33** **a** Charge/discharge profiles of the Zn//I_2_-CFs full cell at the current density of 0.5 C and **b** their corresponding dQ/dV curves


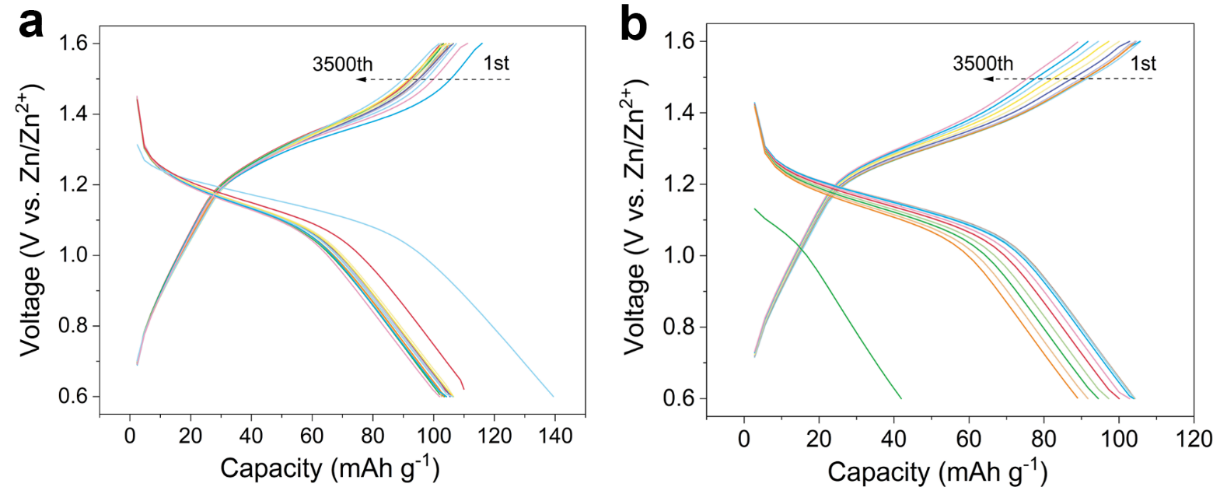


**Fig. S34** Charge/discharge profiles of **a** the Zn//I_2_-CFs and **b** ZnIn//I_2_-CFs full cells at the current density of 5.0 C

**
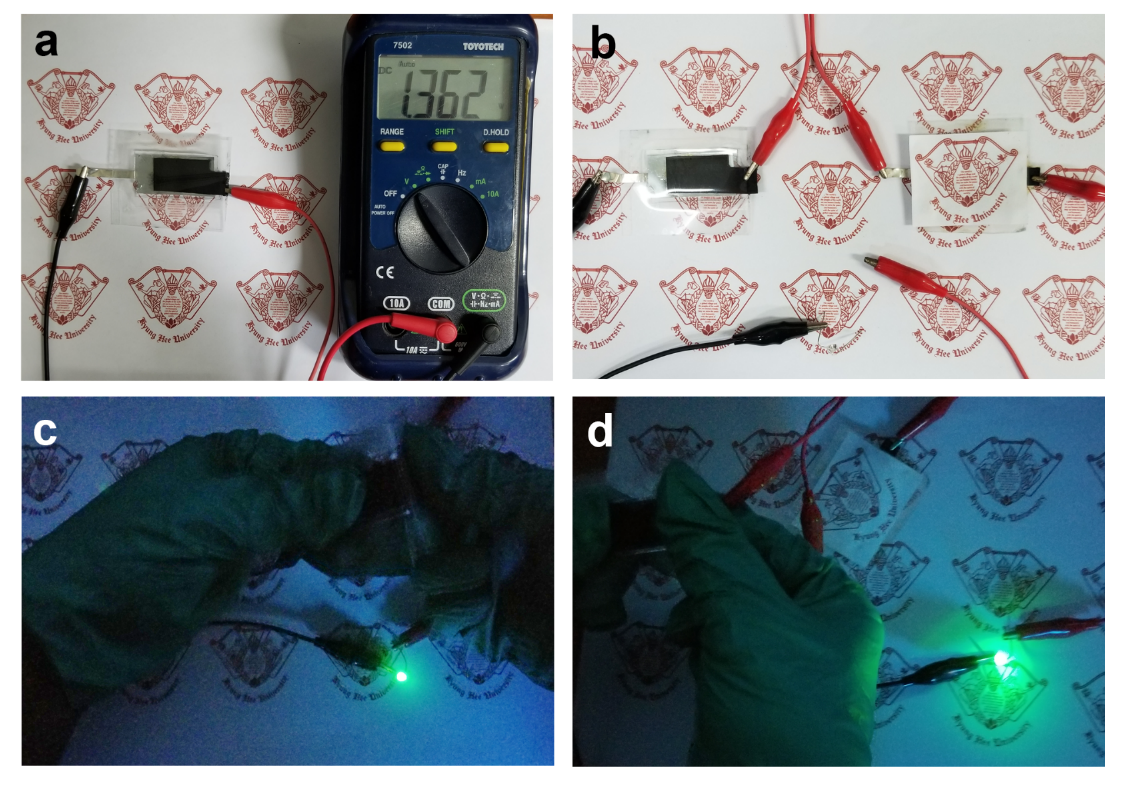
**

**Fig. S35** **a** Open circuit voltage, **b** two cells connected in series, and **c, d** flexibility testing of the quasi-solid-state ZnIn//I_2_-CFs pouch cell

**
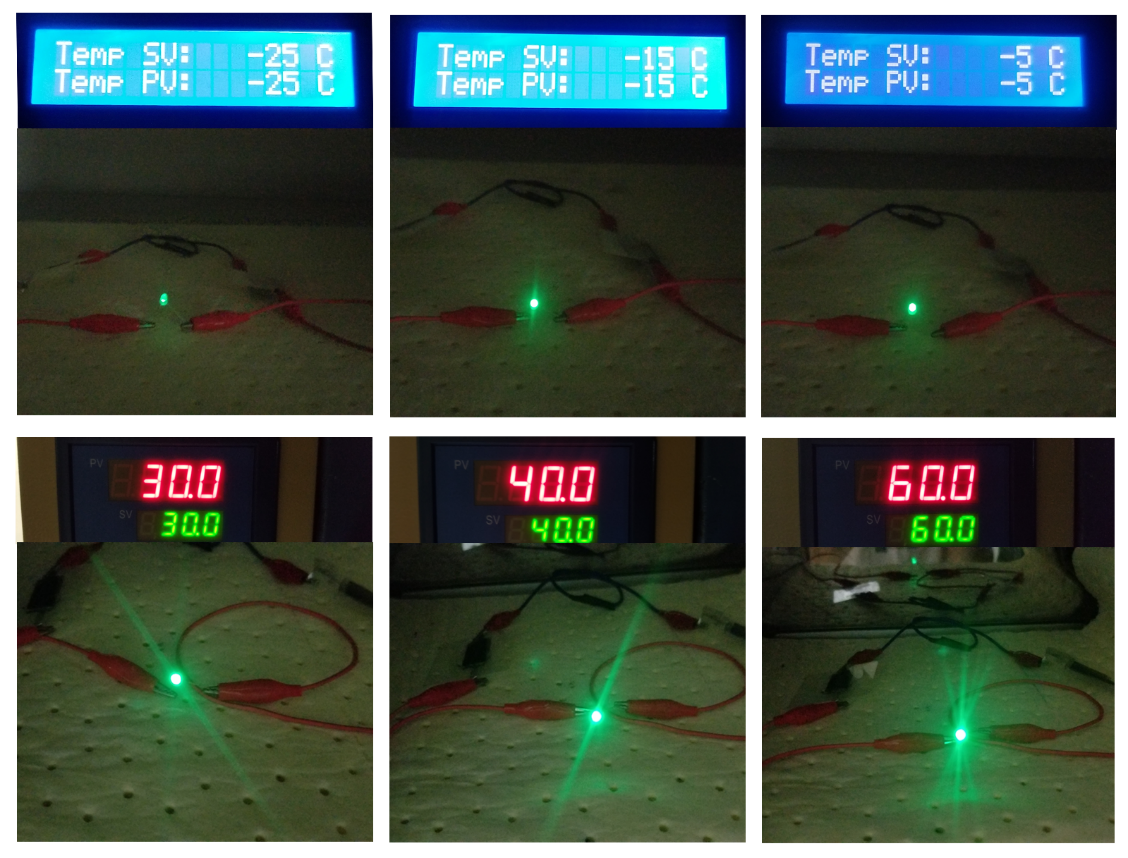
**

**Fig. S36** Optical photographic images of the quasi-solid state ZnIn//I_2_-CFs pouch cell powering a LED under different temperature conditions

**Supplementary References**

[S1] Evans, J., Vincent, C. A. & Bruce, P. G. Electrochemical measurement of transference numbers in polymer electrolytes. Polymer **28**, 2324-2328 (1987). <https://doi.org/10.1016/0032-3861(87)90394-6>

[S2] X. Yang, W. Li, Z. Chen, M. Tian, J. Peng et al., Synchronous dual electrolyte additive sustains Zn metal anode with 5600 h lifespan. Angew. Chem. Int. Ed. **135**, 202218454 (2023). <https://doi.org/10.1002/ange.202218454>

[S3] H. Gan, J. Wu, F. Zhang, R. Li, H. Liu, Uniform Zn2+ distribution and deposition regulated by ultrathin hydroxyl-rich silica ion sieve in zinc metal anodes. Energy Stor. Mater. **55**, 264-271 (2023). <https://doi.org/10.1016/j.ensm.2022.11.044>

[S4] J. Zheng, Z. Cao, F. Ming, H. Liang, Z. Qi et al., Preferred orientation of TiN coatings enables stable zinc anodes. ACS Energy Lett. **7**, 197-203 (2021). <https://doi.org/10.1021/acsenergylett.1c02299>

[S5] Y. An, Y. Tian, S. Xiong, J. Feng, Y. Qian, Scalable and controllable synthesis of interface-engineered nanoporous host for dendrite-free and high rate zinc metal batteries. ACS Nano **15**, 11828-11842 (2021). <https://doi.org/10.1021/acsnano.1c02928>

[S6] S. Jiao, J. Fu, M. Wu, T. Hua, H. Hu, Tailoring Zn^2+^ desolvation kinetics and flux toward dendrite-free metallic zinc anodes. ACS Nano **16**, 1013-1024 (2021). <https://doi.org/10.1021/acsnano.1c08638>

[S7] S. Zhang, M. Ye, Y. Zhang, Y. Tang, X. Liu et al., Regulation of ionic distribution and desolvation activation energy enabled by in situ zinc phosphate protective layer toward highly reversible zinc metal anodes. Adv. Funct. Mater. **33**, 2208230 (2023). <https://doi.org/10.1002/adfm.202208230>

[S8] L. Zhang, J. Huang, H. Guo, L. Ge, Z. Tian et al., Tuning ion transport at the anode-electrolyte interface via a sulfonate-rich ion-exchange layer for durable zinc-iodine batteries. Adv. Energy Mater. **13**, 2203790 (2023). <https://doi.org/10.1002/aenm.202203790>

[S9] J. Shin, J. Lee, Y. Kim, Y. Park, M. Kim et al., Grain-directed zinc deposition in aqueous zinc ion batteries. Adv. Energy Mater. **11**, 2100676 (2021). <https://doi.org/10.1002/aenm.202100676>

[S10] H. Ren, S. Li, B. Wang, Y. Zhang, T. Wang et al., Molecular-crowding effect mimicking cold-resistant plants to stabilize the zinc anode with wider service temperature range. Adv. Mater. **35**, 2208237 (2023). <https://doi.org/10.1002/adma.202208237>

[S11] P. Wang, S. Liang, C. Chen, X. Xie, J. Chen et al., Spontaneous construction of nucleophilic carbonyl-containing interphase towards ultra-stable zinc metal anodes. Adv. Mater. **34**, 2202733 (2022). <https://doi.org/10.1002/adma.202202733>

[S12] P. Xue, C. Guo, L. Li, H. Li, D. Luo et al., A MOF-derivative decorated hierarchical porous host enabling ultrahigh rates and superior long-term cycling of dendrite-free Zn metal anodes. Adv. Mater. **34**, 2110047 (2022). <https://doi.org/10.1002/adma.202110047>

[S13] R. Zhao, X. Dong, P. Liang, H. Li, T. Zhang et al., Prioritizing hetero-metallic interfaces via thermodynamics inertia and kinetics zincophilia metrics for tough Zn-based aqueous batteries. Adv. Mater. **35**, 2209288 (2023). <https://doi.org/10.1002/adma.202209288>

[S14] Y. Li, X. Peng, X. Li, H. Duan, S. Xie et al., Functional ultrathin separators proactively stabilizing zinc anodes for zinc-based energy storage. Adv. Mater. **35**, 2300019 (2023). <https://doi.org/10.1002/adma.202300019>

[S15] Z. Xing, Y. Sun, X. Xie, Y. Tang, G. Xu et al., Zincophilic electrode interphase with appended proton reservoir ability stabilizes Zn metal anodes. Angew. Chem. Int. Ed. **135**, 202215324 (2023). <https://doi.org/10.1002/anie.202215324>

[S16] X. Luan, L. Qi, Z. Zheng, Y. Gao, Y. Xue et al., Step by step induced growth of zinc-metal interface on graphdiyne for aqueous zinc-ion batteries. Angew. Chem., Int. Ed. 2023, **62**, 202215968 (2023). <https://doi.org/10.1002/anie.202215968>

[S17] H. Liu, J. Wang, W. Hua, L. Ren, H. Sun et al., Navigating fast and uniform zinc deposition via a versatile metal-organic complex interphase. Energy Environ. Sci. **15**, 1872-1881 (2022). <https://doi.org/10.1039/d2ee00209d>

[S18] R. Zhao, H. Wang, H. Du, Y. Yang, Z. Gao et al., Lanthanum nitrate as aqueous electrolyte additive for favourable zinc metal electrodeposition. Nat. Commun. **13**, 3252 (2022). <https://doi.org/10.1038/s41467-022-30939-8>

[S19] Q. Li, A. Chen, D. Wang, Y. Zhao, X. Wang et al., Tailoring the metal electrode morphology via electrochemical protocol optimization for long-lasting aqueous zinc batteries. Nat. Commun. **13**, 3699 (2022). <https://doi.org/10.1038/s41467-022-31461-7>

[S20] P. Zou, R. Zhang, L. Yao, J. Qin, K. Kisslinger, et al., Ultrahigh-rate and long-life zinc-metal anodes enabled by self-accelerated cation migration. Adv. Energy Mater. **11**, 2100982 (2021). <https://doi.org/10.1002/aenm.202100982>

[S21] H. Yu, D. Chen, Q. Li, C. Yan, Z. Jiang et al., In situ construction of anode-molecule interface via lone-pair electrons in trace organic molecules additives to achieve stable zinc metal anodes. Adv. Energy Mater. **13**, 20300550 (2023). <https://doi.org/10.1002/aenm.202300550>

[S22] Q. Hu, J. Hou, Y. Liu, L. Li, Q. Ran et al., Modulating zinc metal reversibility by confined antifluctuator film for durable and dendrite-free zinc ion batteries. Adv. Mater. **35**, 2303336 (2023). <https://doi.org/10.1002/adma.202303336>
